# A gene-based capture assay for surveying patterns of genetic diversity and insecticide resistance in a worldwide group of invasive mosquitoes

**DOI:** 10.1371/journal.pntd.0010689

**Published:** 2022-08-08

**Authors:** Matthew L. Aardema, Michael G. Campana, Nicole E. Wagner, Francisco C. Ferreira, Dina M. Fonseca

**Affiliations:** 1 Department of Biology, Montclair State University, Montclair, New Jersey, United States of America; 2 Sackler Institute for Comparative Genomics, American Museum of Natural History, New York, New York, United States of America; 3 Center for Conservation Genomics, Smithsonian’s National Zoo and Conservation Biology Institute, Washington, DC, United States of America; 4 Center for Vector Biology, Rutgers University, New Brunswick, New Jersey, United States of America; Centers for Disease Control and Prevention, UNITED STATES

## Abstract

Understanding patterns of diversification, genetic exchange, and pesticide resistance in arthropod disease vectors is necessary for effective population management. With the availability of next-generation sequencing technologies, one of the best approaches for surveying such patterns involves the simultaneous genotyping of many samples for a large number of genetic markers. To this end, the targeting of gene sequences of known function can be a cost-effective strategy. One insect group of substantial health concern are the mosquito taxa that make up the *Culex pipiens* complex. Members of this complex transmit damaging arboviruses and filariae worms to humans, as well as other pathogens such as avian malaria parasites that are detrimental to birds. Here we describe the development of a targeted, gene-based assay for surveying genetic diversity and population structure in this mosquito complex. To test the utility of this assay, we sequenced samples from several members of the complex, as well as from distinct populations of the relatively under-studied *Culex quinquefasciatus*. The data generated was then used to examine taxonomic divergence and population clustering between and within these mosquitoes. We also used this data to investigate genetic variants present in our samples that had previously been shown to correlate with insecticide-resistance. Broadly, our gene capture approach successfully enriched the genomic regions of interest, and proved effective for facilitating examinations of taxonomic divergence and geographic clustering within the *Cx*. *pipiens* complex. It also allowed us to successfully survey genetic variation associated with insecticide resistance in *Culex* mosquitoes. This enrichment protocol will be useful for future studies that aim to understand the genetic mechanisms underlying the evolution of these ubiquitous and increasingly damaging disease vectors.

## Author summary

The mosquito taxa that make up the *Culex pipiens* complex are important vectors of the agents of several human diseases such as West Nile and St. Louis encephalitides, and lymphatic filariasis. They are also important vectors of avian malaria, which impacts livestock and wildlife. The development of effective strategies for the control of these mosquitoes requires knowledge of their origins, distribution, dispersal patterns, and the extent to which discreet taxonomic entities within the complex interbreed. To achieve these objectives, it is necessary to compare patterns of genetic diversity across many mosquito samples, which can be cost-prohibitive. To address this limitation, we developed a targeted, gene-based assay that allowed us to cost-effectively genotype a large number of genetic variants from a representative global sampling of individual *Cx*. *pipiens* complex mosquitoes. We show that this assay is a powerful tool for examining genetic structure and hybridization among populations. We also explore its utility for surveying alleles previously shown to be associated with insecticide resistance. Future use of this enrichment assay and the bioinformatics methods described here will allow researchers to study evolutionary patterns across the *Cx*. *pipiens* complex as well as monitor the presence of genetic variation that could affect control efforts.

## Introduction

The brown, dusk-biting mosquitoes collectively classified within the *Culex pipiens* complex (Diptera: Culicidae), include two globally distributed invasive species, the temperate *Culex pipiens*, and the tropical *Cx*. *quinquefasciatus*, along with several additional taxa with more restricted distributions [1]. Specific populations of these two species are critical urban vectors of the nematode that causes human periodic filariasis (*Wuchereria bancrofti*), and several epidemic encephalitides such as West Nile virus [2] and Usutu virus [3]. These mosquitoes also vector avian malaria, a group of parasites that are of significant concern to island bird communities in Hawaii, the Galapagos, and elsewhere [4–7].

Rapid human movements around the globe likely facilitated the spread of many now cosmopolitan mosquito species such as several in the *Cx*. *pipiens* complex, and accordingly these distributions are a relatively recent phenomenon [8]. One of the best-studied invasive species is the yellow fever mosquito, *Aedes aegypti*. Outside its source location in Africa, populations of *Ae*. *aegypti* all share the same basic genotype, revealing its rapid, human-facilitated expansion [9]. Interestingly, in contrast to this pattern, microsatellite analyses of populations of *Cx*. *pipiens* and *Cx*. *quinquefasciatus* from across the world have uncovered unexpectedly high levels of genetic diversity. For example, continental populations of *Cx*. *quinquefasciatus* flanking the Pacific Ocean are highly differentiated [10]. Furthermore, although historical records pinpoint an original introduction of *Cx*. *quinquefasciatus* into the Hawaiian Islands from the Americas [11], current Hawaiian *Cx*. *quinquefasciatus* have a distinct Australasian signature [10]. The mechanisms underlying the likely replacement of the first population in Hawaii by the second are unknown and understanding this process will require a better understanding of the specific genetic makeup (i.e., which genes and their capabilities) of the population(s) involved.

Another important aspect of the *Cx*. *pipiens* complex is the extent to which genetic exchange (hybridization) has contributed to ecological divergence and patterns of disease transmission. For example, inter-taxonomic hybridization between the two forms of *Cx*. *pipiens* may have significant negative consequences for arboviral transmission to humans [12]. Several studies have also found evidence of extensive hybrid zones between temperate *Cx*. *pipiens* or *Cx*. *pipiens pallens* (a subspecies limited to northeastern Asia) and tropical *Cx*. *quinquefasciatus* [13,14]. Finally, analysis of genetic variation at the acetylcholinesterase locus 2 (ACE2) across members of the complex indicated that the hybridization event that may have resulted in formation of the temperate *Cx*. *pipiens pallens* was unidirectional which is surprising since patterns of hybridization of contemporary *Cx*. *p*. *pallens* with *Cx*. *quinquefasciatus* appear bidirectional [13].

To address these and other questions specific to the *Cx*. *pipiens* complex, it will be necessary to extensively survey population and taxonomic samples at a large number of independently segregating molecular markers. Such an analysis would provide greater clarity to patterns of evolutionary divergence, global movement, and genetic exchange within these mosquitoes. Next-generation sequencing (NGS) has enabled vast amounts of genetic data to be collected at relatively low cost [15,16]. However, challenges for sample-specific data collection and analysis are created by the presence of diverse microbial symbionts such as *Wolbachia* and endogenous viral elements in these mosquitoes [17]. Furthermore, mosquito genomes like those of *Culex* are often riddled with repetitive DNA [18]. Of a recent assembly of the 567.56 Mb *Cx*. *pipiens pallens* genome, 60.63% (344.11 Mb) was found to consist of repetitive elements [19]. Such elements make whole genome data collection and analysis expensive and wasteful since only a small proportion of the genetic variation observed can be confidently compared across all specimens.

Capitalizing on recent technological advancements, a capture approach where DNA or RNA probes designed to match known genes are hybridized to DNA libraries of individual specimens and sequenced has been gaining traction [20–22]. Because it bypasses large amounts of DNA of unknown function and heritability, targeted gene enrichment allows users to pool tens or even hundreds of indexed specimens, and cost-effectively sequence thousands of homologous loci simultaneously. However, such enrichment methodologies have so far been minimally applied in mosquitos for examining population genetics or evolutionary patterns (but see [23]).

Here we describe the design and use of a genetic baits assay targeting 512 genes annotated in the *Cx*. *quinquefasciatus* genome including regions that have been shown to harbor genetic variation that correlates with insecticide resistance. We examined the utility of these baits for taxonomic differentiation and patterns of admixture by sequencing samples from four taxa of the *Cx*. *pipiens* species complex, samples of known hybrid origin, and one sample of a closely related, outgroup taxon, *Culex torrentium*. To further examine the potential of these baits for exploring finer scale, intra-taxonomic population structure and differentiation, we included samples of *Cx*. *quinquefasciatus* from multiple geographic sources. Finally, within our samples we investigated the presence and frequency of alleles previously found to correlate with insecticide resistance. This was done to test the utility of these baits for surveying genetic variation that may contribute to a reduced efficacy of chemical control efforts. Such information can be critical for developing effective strategies to mitigate disease transmission by these mosquitoes [24].

## Methods

### Bait design and screening

We designed an in-solution capture assay targeting 131 rapidly evolving *Culex* genes obtained from a previous comparison of *de novo-*assembled transcriptomes from multiple samples of *Cx*. *pipiens* f. pipiens and *Cx*. *pipiens* f. molestus [25]. These ‘rapidly evolving’ genes were enriched for seven GO terms, of which five terms (chitin metabolic process, chitin binding, serine-type endopeptidase activity, proteolysis and odorant binding) were also enriched along the ‘fly’ branch [26]. This indicates they may represent a genetic ‘core’ for adaptive evolution within the Diptera. To facilitate estimates of genotyping error rates, we also included 28 identified ‘slow-evolving’ genes [25]. To these 131 rapidly evolving and 28 slow evolving genes, we also added 353 genes potentially involved in insecticide resistance. These included annotated P450s, alpha and beta esterases, sodium channel genes, and acetylcholinesterase genes [27]. In total, our capture assay targeted 512 genes (S1 Table). These genes were then extracted from the *Cx*. *quinquefasciatus* genome (v. CpipJ2.5) [28] using their VectorBase annotations (https://vectorbase.org/vectorbase/app) [29].

To ensure optimal enrichment, we commissioned Daicel Arbor Biosciences (https://arborbiosci.com/) to design 39,953 120 bp baits with ~1.5x flexible tiling density (~80bp probe spacing) across our targeted genes. These baits covered the complete exonic and intronic regions for each gene, allowing for simultaneous investigation of both adaptive and neutral evolution. These candidates were then assessed using BLAST v. 2.12.0 [30]. Bait candidates were accepted when they satisfied one of the following conditions: a) no BLAST hit with a melting temperature (T_m_) above 60°C, b) no more than two hits at T_m_ 62.5–65°C, or 10 hits in the same interval and at least one neighbor candidate being rejected. c) no more than 2 hits at T_m_ 65–67.5°C and 10 hits at T_m_ 62.5–65°C and two neighbor candidates on at least one side being rejected. d) no more than a single hit at or above T_m_ 70°C or e) no more than one hit at T_m_ 65–67.5°C and 2 hits at T_m_ 62.5–65°C and two neighbor candidates on at least one side being rejected. The baits were synthesized as a myBaits version 3 kit. After stringent filtration, 29,992 baits were retained, covering all 512 target genes with at least one bait. The targeted sequences total 2,524,269 bp in length, and are well distributed across the three *Cx*. *quinquefasciatus* chromosomes (S1 Fig).

### Target enrichment and sample sequencing

To test our targeted enrichment approach, we chose specimens representative of the genetic diversity observed across the complex (S2 Table). Specifically, we included specimens of the two *Culex pipiens* forms from Europe and North America (f. pipiens and f. molestus), specimens of the subspecies *Cx*. *pipiens pallens* from the Republic of Korea and, to assess the power of the assay to discern intraspecific patterns of diversity, specimens of *Cx*. *quinquefasciatus* from six distinct geographic regions: east-southeast Asia, Samoa, Hawaii, North America (including the Caribbean), Brazil and Nigeria. We also included known hybrids of *Cx*. *pipiens* and *Cx*. *quinquefasciatus* from California and North Carolina. Most specimens had previously been examined using a panel of microsatellite loci [10,12,13,31]. Finally, we included one sample of the closely related species *Cx*. *torrentium* for outgroup comparisons.

We extracted DNA from individual mosquitoes using a phenol-chloroform method previously described [32]. We then performed an initial step to clean and concentrate DNA by using Omega Mag-Bind TotalPure NGS beads at 0.9 ratio following the manufacturer’s protocol. For library preparation, we used the Illumina DNA library prep (formerly Nextera DNA Flex), again following the manufacturer’s protocol. Each sample was given a unique, barcoded adapter in this step to allow library multiplexing prior to sequencing. DNA concentration and quality of the libraries were determined using the Qubit 2.0 Fluorometer and Bioanalyzer High Sensitivity DNA Analysis kit (Agilent), respectively. To create amplicons that did not have affinity to streptavidin, we performed four amplification cycles following instructions in Appendix A2 of the myBaits Hybridization Capture for NGS protocol (v. 4.01). To do this, we used universal P5 and P7 primers. The resulting products were cleaned using Omega Mag-Bind beads and hybridized with our capture biotinylated baits for target enrichment following myBaits protocol (v. 4.01). We used diluted baits to a ratio of 1:6. These libraries were amplified following 12 cycles using KAPA HiFi Hotstart ready mix, and the resulting products were cleaned with AMPure XP beads (Beckman Coulter). Concentration and quality of final libraries were checked using Qubit and Bioanalyzer, and each sample was adjusted to a final concentration of 4 nM (1.33 ng/μl). We obtained libraries with fragment sizes of 600 bp on average. These were 2 × 300 bp paired-end sequenced in multiplexed groups of six or seven samples on an Illumina MiSeq using 600-cycle MiSeq version 3 kits.

### Data mapping and variant calling

After sequencing, we first used Trim Galore v. 0.4.1 [33] to trim Illumina sequencing adapters and bases from read ends with a quality score less than 20 (Cutadapt version 1.9.1) [34]. We removed both reads of a pair if either was less than 30 bases long after trimming. We mapped all remaining trimmed reads to the *Cx*. *quinquefasciatus* reference genome (v. CpipJ2.5) [28] using BWA-MEM v. 0.7.12 with default settings [35]. Next, we added read groups and sorted the mapped reads using the AddOrReplaceReadGroups function in Picard v. 1.119 [36]. We then marked read duplicates using the tool MarkDuplicates, also with Picard v. 1.119, followed by indel realignment using IndelRealigner in the Genome Analysis Toolkit (‘GATK’) v. 3.6 [37]. Finally, for each sample, we identified genetic variants using GATK’s HaplotypeCaller [38] (specific flags:--emitRefConfidence GVCF,--variant_index_type LINEAR,--variant_index_parameter 128000 -rf BadCigar).

With the resulting raw VCF files (one per sample), we used GATK’s GenotypeGVCFs function to produce a single, multi-sample VCF containing all identified variants observed across all samples. This file was filtered to retain only single nucleotide polymorphisms (SNPs), using the SelectVariants tool in GATK v. 4.0.8.1 [39]. This tool was also used to remove any variants that fell outside our designated baits coordinates (S1 Table). Next, we applied a series of hard quality filters, removing all SNPs with any of the following parameters: QD < 11.0, FS > 40.0, MQ < 56.0, MQRankSum < -0.2, ReadPosRankSum < -3.0, and/or SOR > 2.0. These thresholds were based on the observed distribution of variants (S2 Fig), and were equal to, or more stringent than, the recommended values given in GATK’s best practices [40]. Finally, we used SnpEff v. 4.3 [41], with a custom database to annotate the remaining SNPs for downstream sorting by variant type.

We did not sequence any unenriched libraries in parallel with our enriched library sequencing efforts. However, for the purpose of comparing the enrichment efficiency of our bait capture assay to unenriched libraries, we used previously published Illumina data from two *Cx*. *pipiens* f. pipiens, two *Cx*. *pipiens* f. molestus, and one *Cx*. *pipiens pallens* (S3 Table). These data were generated using similar methods to those used here, but without the application of any enrichment method [19,42]. Four of these five datasets were prepared from single, wild-caught mosquitoes [42], while the fifth was a pool of laboratory-maintained samples [19]. As each dataset contained substantially more reads than what we obtained from our capture-assay libraries, we used the program Seqtk v. 1.1-r91 [43], to down sample each dataset’s reads to three million pairs (after trimming and quality filtering). After read down sampling, we mapped the reads, sorted them, and realigned INDELs as described above. These data were not included in our subsequent clustering analyses nor in our insecticide resistance investigation. For all datasets (both enriched and unenriched), the ‘stats’ function in SAMtools v. 1.15 [44] was used to determine the number of properly paired reads that mapped to the full genome, the number of properly paired reads that mapped to our target regions, and the percentage of target regions with a depth of coverage equal to or greater than three reads (≥3×).

### Genetic clustering and admixture

In addition to examining the enrichment efficiency of our bait capture approach, we also wanted to assess our enriched dataset’s utility for surveying inter-taxonomic relationships and potential gene flow (admixture) across samples derived from the *Cx*. *pipiens* species complex, as well as for surveying intraspecific population relationships. As prior work has shown the importance of using a large number of segregating markers to detect structure from genetic data when divergence between distinct populations is likely to be low [45], we wanted to maximize the number of selectively neutral markers used. Therefore, we selected all variants that were annotated as either ‘synonymous’ or ‘intronic’, as they are more likely to be “neutral”. Although research in *Drosophila* suggests that mutations in both of these site categories can experience selection [46–48], the strength of this selection is likely far less than that acting on non-synonymous variation.

We used GATK’s ‘SelectVariants’ tool to generate two new VCFs from our VCF database of high quality synonymous and intronic SNPs, one with all samples except the outgroup *Cx*. *torrentium* (henceforth ‘*Cx*. *pipiens* complex’ dataset), and a second with only the *Cx*. *quinquefasciatus* samples (henceforth ‘*Cx*. *quinquefasciatus*’ dataset). We then removed any variant from both datasets that was not in Hardy-Weinberg equilibrium (p < 0.0001), and any variant in which the minor allele was represented at less than 5% frequency. Both filtering steps were carried out using VCFtools v. 0.1.17 [49]. Finally, for both datasets, we used PLINK v.1.90b6.6 [50] to remove SNPs with a pairwise squared correlation (r^2^) greater than 50% within sliding windows of 50 SNPs at 10 SNP increments between windows [51]. This was done to reduce the impact of linkage between SNPs on our examinations of population clustering and admixture [52].

We first used principal component analyses (PCAs) to investigate non-parametric clustering among the samples in both datasets. These PCAs were conducted with the program PLINK v. 1.90b6.6 [50], and the results visualized using R v. 4.0.2 [53], focusing on the first two principal components (PC1 & PC2). We also examined patterns of genetic structure within our data using a Discriminant Analysis of Principal Components (DAPC) [54], as well as a maximum likelihood approach with the program ADMIXTURE v. 1.3.0 [55]. The DAPC were carried out with the package *adegenet* v. 2.1.5 [56] in R. We first used the ‘find.clusters’ function to identify probable genetic clusters represented in the data, For this analysis, we retained all principal components. To determine the optimal number of clusters (K), we used the Bayesian information criterion (BIC) [57]. If our BIC results indicated the optimal number of clusters was greater than one, then the number of retained principal components was determined by using the ‘cross-validation’ function in *adegenet*, with sample assignments determined in the initial clustering analysis. We used 75% of the data for a training set and the remaining 25% for data confirmation. This was repeated for 100 replicates. We used the ‘dapc’ function to probabilistically assign each sample to a cluster. From the information on discriminant functions, a genotype composition plot (Compoplot) was generated indicating the attributed probabilities of each sample to a cluster [56].

With ADMIXTURE, we examined potential clusters (K) from one to seven in both datasets. Each K value was run 20 independent times with a different seed value for each run. Across K values, we compared the means observed for the standard error of the 10-fold cross-validation (CV) error estimate to determine the number of clusters best supported by the data [58]. We determined the average q-matrix cluster assignment for each sample for each K value using the online version of CLUMPAK [59], with default settings.

### Genetic diversity and taxonomic divergence

To examine the amount of genetic diversity harbored within individual samples, populations, and taxa, we used GATK v. 4.0.8.1 [39] to designate all sample-variant combinations with a depth of coverage less than 15× as a ‘no call’. A read depth of 15× or greater has been shown to be adequate for assessing the diploid state of an allele (homozygous vs. heterozygous) within a sample with potentially high amounts of heterogeneity [60]. No upper limit was placed on read depth. Next, we used GATK to retain only biallelic SNPs that were annotated as either ‘synonymous’ or ‘intronic’ and called in all samples. This variant filtering was done to improve the equivalency of relative diversity estimates across all the samples. Finally, we used VCFtools v. 0.1.17 [49] to count the number of observed homozygous variants. The resulting data were used to calculate the average heterozygosity within a sample across assessed sites [61,62]. We also calculated taxon and population (*Cx*. *quinquefasciatus* only) means and standard errors of the means. Although these estimates do not give us absolute estimates of genetic diversity (because they only include known segregating sites), they do allow us to make relative comparisons between groups of samples (e.g., taxa or populations).

To examine relative divergence between sample clusters (e.g., taxa or populations), we used VCFtools v. 0.1.17 and our larger clustering dataset to calculate the pairwise fixation index (F_st_) [63]. Comparisons were done between the four complex taxa excluding the known hybrids, and between these and the outgroup *Cx*. *torrentium*. We also compared the *Cx*. *quinquefasciatus* populations from the six designated geographic regions. All sample taxonomic and population designations were based on their prior assignments (S2 Table). We report both the weighted and unweighted estimates. Weighted estimates may be more strongly impacted by unequal sample sizes, whereas unweighted estimates may be more affected by variants segregating at low frequencies [64].

### Phylogenetic analysis

To further examine sample clustering as well as taxonomic relationships amongst all samples, including the outgroup *Cx*. *torrentium*, we performed a maximum likelihood phylogenetic analysis. For this analysis, we focused on neutral variants that are likely to have similar mutation probabilities. Therefore, from our annotated variants dataset, we used BCFtools v. 1.9 [65] to select only 4-fold (‘silent’) segregating sites. Next, we removed variants that were not in Hardy-Weinberg equilibrium using VCFtools v. 0.1.17. We also thinned highly correlated SNPs as described above. The resulting VCF file was converted to PHYLIP format using the vcf2phylip.py v. 1.5 python script [66]. We then used jModelTest 2.1.10 [67,68] with default settings to select the best-fit model of nucleotide substitution for our datasets based on BIC scores. With the best fitting model, we used PhyML v. 3.1 [69] to carry out a maximum-likelihood phylogenetic analysis, with 100 non-parametric bootstrap replicates to determine confidence values. The resulting phylogenetic tree was visualized using the program FigTree v. 1.4.4 [70].

### Presence of variants potentially conferring insecticide resistance

To assess the utility of our capture assay for surveying genetic polymorphism that may contribute to insecticide resistance, we first conducted a literature survey to identify known single nucleotide variants that have been shown to be associated with insecticide resistance in *Cx*. *pipiens* complex mosquitoes. Specifically, we examined publications that reported the gene and position of a segregating variant that correlated with resistance to one or more active insecticidal products (e.g., organophosphates or pyrethroids). These were exclusively missense mutations that changed the amino acid sequence and likely protein interactions with the insecticide. With their genome coordinates (chromosome and base position), we used VCFtools v. 0.1.17 to calculate the frequencies of the susceptible and resistant alleles across all our samples. We also used VCFtools to examine the sample-specific presence of these variants to compare taxa and populations.

## Results

### Data mapping and variant calling

The average percentage of total reads mapped to the full genome was very similar between our enriched libraries prepared with a capture assay and unenriched libraries (79.8% vs. 81.3%; Table 1). However, the enriched libraries had an average of 13.82% of the reads mapped to the target gene regions, whereas the unenriched datasets only had 0.76%. This indicates an enrichment factor of 18.2 fold for the target regions. A difference between the enriched and unenriched data was also reflected in the percentage of the target regions covered by three or more reads (Table 1).

**Table 1:** Comparison of read-mapping between enriched and unenriched libraries. We report the average number of read pairs after quality trimming, the number of read pairs that properly mapped to the full genome, and the percent of the reads which mapped to the full genome. The standard deviations are given in parentheses. Also included are the number of read pairs that mapped to the target bait regions, the percentage of the properly paired reads that mapped to the target regions, and the percentage of the target bait regions with a coverage of three or more reads (≥3×). For individual sample statistics see S3 Table.

	Post-trimming Read Pairs	Properly Paired Reads Mapped to the Full Genome	% all reads mapped	Properly Paired Mapped Reads (Target Regions)	% Properly paired reads mapped to target regions	% of target regions with coverage ≥3x
**Enriched Datasets (n = 36)**	2,504,426 (1,289,201)	2,036,689 (1,198,782)	79.8% (11.8%)	274,708 (146,076)	13.82% (2.27%)	52.6% (14.27%)
**Unenriched Datasets (n = 5)**	3,000,000 (0*)	2,439,858 (88,964)	81.3% (3.0%)	18,562 (872)	0.76%. (0.01%)	10.65% (1.19%)

^*^For each unenriched dataset, the number of reads used was down sampled to three million after read trimming and quality filtering. See text for more details.

We initially called 12,301,010 variants across all samples, including both single nucleotide polymorphisms (SNPs) and insertions/deletions (INDELs). After removing all INDELs and any additional variants not located in our designated baits, we were left with 315,512 SNPs. Quality filtering further reduced this to 132,185 SNPs.

### Genetic clustering and admixture

**Fig 1:**
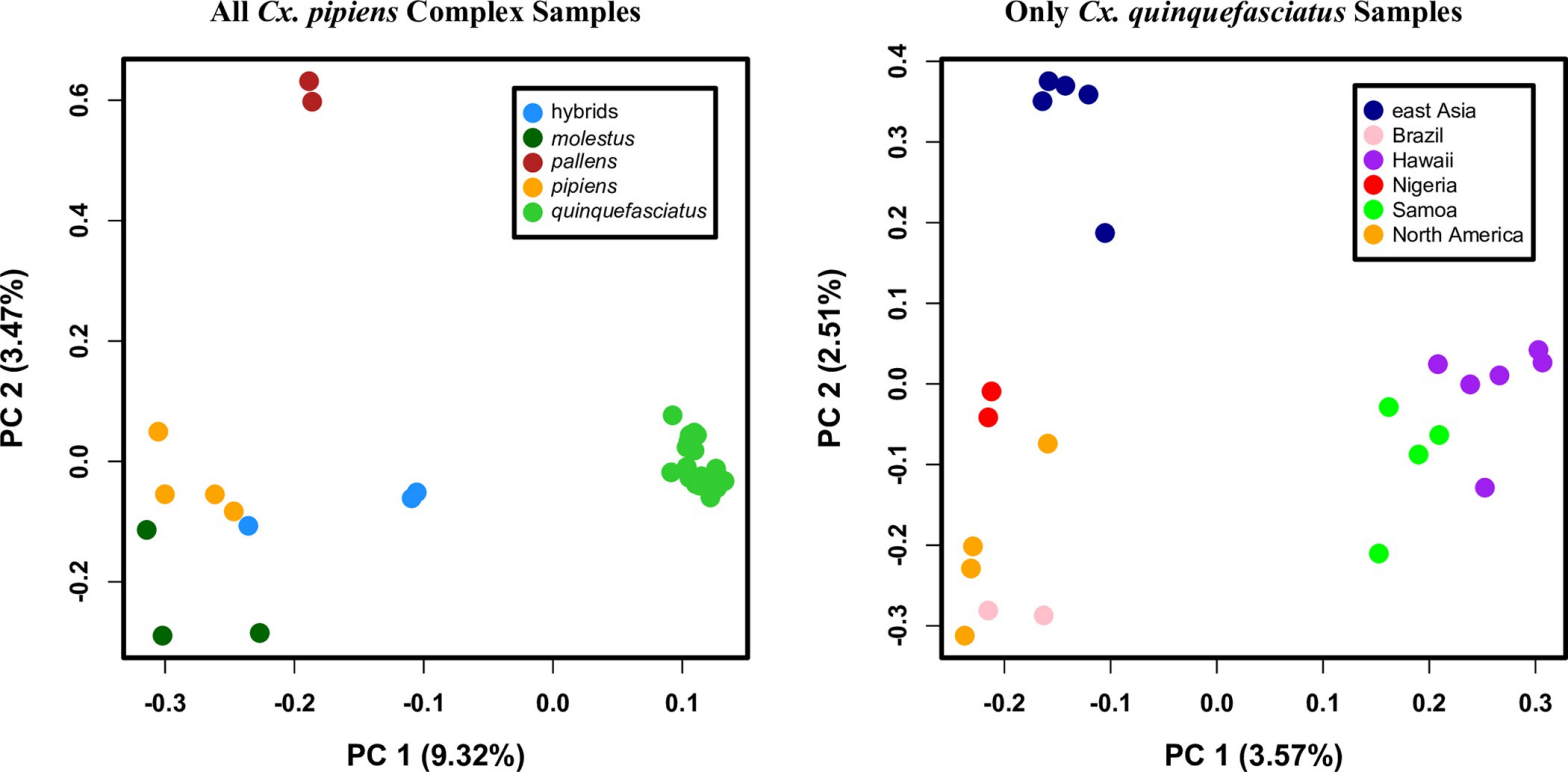
Results of Principal Component Analyses. Shown are the first and second principal components (PC1 & PC2) for all the *Cx*. *pipiens* complex samples (left panel) and just the *Cx*. *quinquefasciatus* samples (right panel). These analyses were performed with neutral, segregating variants. Taxonomic and population memberships were based on prior designations and collection location respectively.

For examining genetic relationships for all the samples within the *Culex pipiens* complex, we generated a dataset consisting of 14,303 unlinked SNPs annotated as either ‘synonymous’ or ‘intronic’. A principal component analysis with this dataset revealed that the greatest genetic divergence (indicated along PC1) occurred between the samples designated as *Cx*. *quinquefasciatus* and all the other samples (Fig 1). PC2 distinguished the *Cx*. *pipiens pallens* samples from the other samples. As expected, the two samples known to be admixed between *Cx*. *quinquefasciatus* and *Cx*. *pipiens* were intermediate between these taxa along PC1. Additionally, along PC2, there appeared a small distinction between the two forms of *Cx*. *pipiens* (f. pipiens and f. molestus), suggesting possible taxonomic-specific genetic divergence. From the DAPC using all complex samples, the most likely number of clusters was K = 2 (BIC = 250.23; S3A Fig). We retained two principal components which accounted for 23.5% of the observed variance. The examination of the first discriminant function showed no overlap between the two clusters (S3B Fig). One cluster was comprised of the *Cx*. *quinquefasciatus* samples, with all other samples in the second cluster (S3C Fig). There was no evidence of admixture in this analysis.

**Fig 2:**
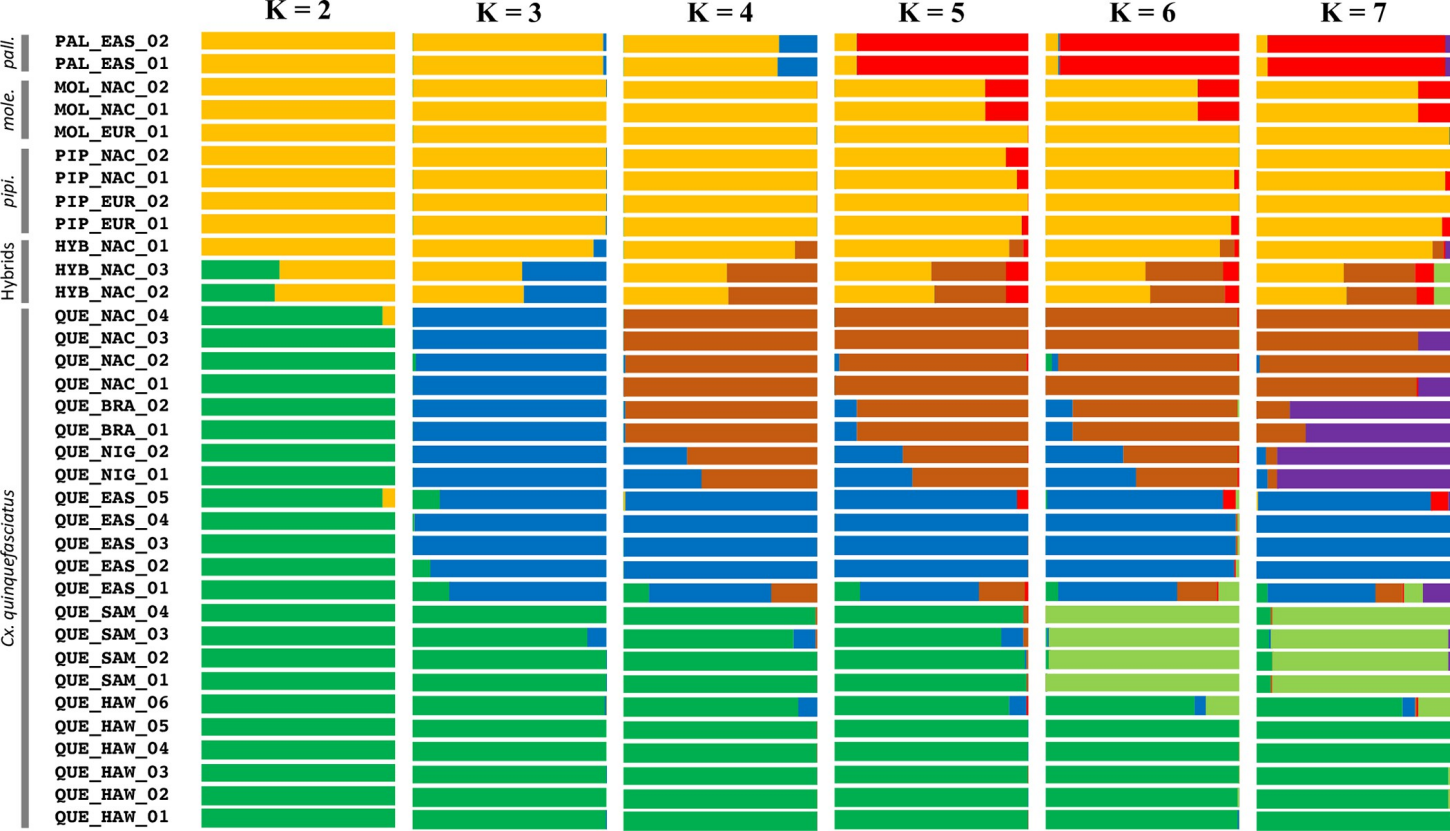
All complex ADMIXTURE results. Shown are the percent ancestry assignments (Q) for K values 2 through 7 based on our analysis of admixture. Sample designations are given on the left along with taxonomic designations.

The analysis of clustering using ADMIXTURE also indicated that a K value of 2 was best supported (S4A Fig). Population clustering at this K value again indicated the genetic distinction between the *Cx*. *quinquefasciatus* samples and the other complex samples (Fig 2). However, the two samples known to be hybrids between *Cx*. *pipiens* and *Cx*. *quinquefasciatus* clearly showed their mixed ancestry. At K = 3 we saw a division between *Cx*. *quinquefasciatus* samples from Hawaii and Samoa and all other *Cx*. *quinquefasciatus* samples. At K = 4 the *Cx*. *quinquefasciatus* samples were further subdivided. At K = 5, the *Cx*. *pipiens pallens* samples were distinguished. Larger K values (6 & 7) further divide the *Cx*. *quinquefasciatus* samples and revealed samples with varying degrees of admixture.

We also looked at sample clustering just in our known *Cx*. *quinquefasciatus* samples. This dataset consisted of 9,829 unlinked, segregating variants annotated as ‘synonymous’ or ‘intronic’. All samples clustered within their known geographic region (Fig 1), and more broadly there were three distinct groupings. These corresponded to a cluster of Hawaiian and Samoan samples that were distinct from all the other samples along PC1, and a cluster of east Asian samples that were distinct from the third cluster along PC2. This third cluster consisted of samples from North America and the Caribbean, Brazil, and Nigeria. The DAPC with just the *Cx*. *quinquefasciatus* samples suggested they derived from a single cluster (K = 1; BIC = 159.48; S3D Fig). This was not surprising given the limited number of markers used and the low amounts of genetic divergence likely to be present among populations of this species [45]. Given this result, we did not perform additional tests within the DAPC analytical framework for this dataset.

**Fig 3:**
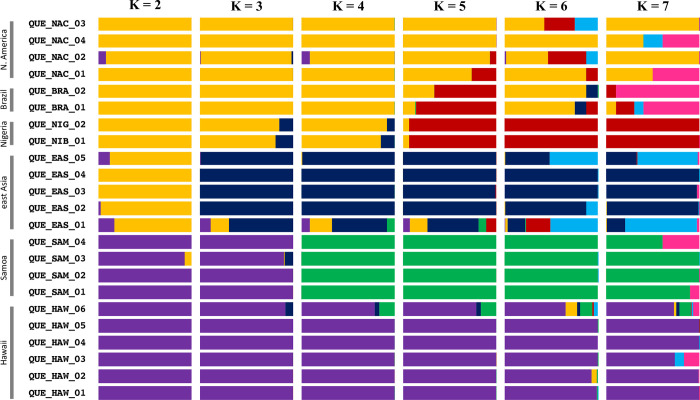
*Cx*. *quinquefasciatus* ADMIXTURE results. Shown are the percent ancestry assignments (Q) for K values 2 through 7 based on our analysis of admixture. Sample designations are given on the left along with population designations.

The admixture results for the *Cx*. *quinquefasciatus* samples also suggested a single taxonomic group (i.e.; K = 1; S4B Fig). However, when we looked at sample clustering at higher K values, we saw the greatest distinction between specimens deriving from Hawaii and Samoa, and all remaining samples (Fig 3). At K = 3 we saw the east Asian samples form a distinct cluster, recapitulating the results for our principal component analysis. One sample from India (QUE_EAS_01), appeared to be highly admixed with genetic representation from multiple populations across K values. At K = 4, the Hawaiian and Samoan samples formed distinct clusters. The Nigerian and Brazilian samples showed their distinctiveness (and relation to one another) at K = 5. However, this affiliation disappeared at K = 6. Such cluster shifting across K values highlights the overall degree of genetic similarity among these samples and likely reveals both a need for larger sample sizes and the limitations of this approach for examining fine-scale structuring between closely related populations in the *Cx*. *pipiens* complex.

### Genetic diversity and taxonomic divergence

To examine relative genetic diversity within all *Cx*. *pipiens* complex mosquitoes sequenced, we used 916 biallelic, neutral SNPs which each had a depth of at least 15 reads (15×) in all samples. The mean number of heterozygous sites and the mean sample pairwise heterozygosity (π) for all taxa are given in Table 2, and each sample’s individual diversity observations are given in S4 Table. The taxon/group with the highest π values was *Cx*. *pipiens pallens* at 0.091 (SE = 0.006). This value means that among the *Cx*. *pipiens pallens*, on average 9.1% of the 916 SNPs were found in a heterozygous state. The next highest value of π was observed in the *Cx*. *torrentium* sample with 0.084. The known hybrids had an average π of 0.066 (SE = 0.009). The lowest mean π value was observed in the various *Cx*. *quinquefasciatus* samples (0.023, SE = 0.002).

**Table 2:** Relative genetic diversity within taxa across 916 neutral, bi-allelic, segregating SNPs. Given are the taxonomic designations (including a category for known hybrid samples), sample size for each taxon, the mean number of heterozygous sites observed per sample with standard error, and the corresponding mean pairwise sample heterozygosity value with standard error.

Taxon	Sample Size (n)	Mean Number of Heterozygous Sites (SE)	Mean Sample Pairwise Heterozygosity (π) (SE)
known hybrids	3	60.7 (7.9)	0.066 (0.009)
*Cx*. *pipiens* f. molestus^a^	3	25.0 (11.0)	0.027 (0.012)
*Cx*. *pipiens* f. pipiens	4	62.3 (7.2)	0.068 (0.008)
*Cx*. *pipiens pallens*	2	83.5 (5.5)	0.091 (0.006)
*Cx*. *quinquefasciatus*	23	20.9 (2.1)	0.023 (0.002)
*Cx*. *torrentium*	1	77	0.084

^**a**^taxon identification was based on examination of male genitalia, geographical source, or in some cases expression of autogeny, as well as prior examination with panels of microsatellite loci (please refer to the text)

To examine relative genetic diversity within just the *Cx*. *quinquefasciatus* samples, we used 540 SNPs that were determined to be biallelic and had a depth of at least 15 reads (15×) in the samples under consideration. These SNPs were also considered most likely to be evolving neutrally by virtue of being annotated as ‘synonymous’ or ‘intronic’. The mean number of heterozygous sites and the mean sample pairwise heterozygosity (π) for the six geographic designations of *Cx*. *quinquefasciatus* are given in Table 3. The samples from east Asia had the highest mean observed π with a value of 0.150 (SE = 0.015). Hawaiian samples also appeared to be relatively genetically diverse with a π value of 0.103 (SE = 0.017). The lowest mean values of π were observed in the Samoan (0.070, SE = 0.013) and Brazilian samples (0.019, SE = 0.012).

**Table 3:** Relative genetic diversity within populations of *Cx*. *quinquefasciatus* across 540 segregating, neutral, bi-allelic SNPs. Given are the population designation, sample size for each population, the mean number of heterozygous sites observed per sample with standard error, and the corresponding mean pairwise sample heterozygosity value with standard error.

Population	Sample Size (n)	Mean Number of Heterozygous Sites	Mean Sample Pairwise Heterozygosity
east Asia	5	81.0 (7.9)	0.150 (0.015)
Samoa	4	38.0 (6.9)	0.070 (0.013)
Hawaii	6	55.5 (8.9)	0.103 (0.017)
North America & Caribbean	4	46.5 (8.5)	0.086 (0.016)
Brazil	2	10.5 (6.5)	0.019 (0.012)
Nigeria	2	48.0 (11.0)	0.089 (0.020)

Table 4 gives the pairwise unweighted and weighted estimates of the fixation index (F_st_) [63], between each of the four *Cx*. *pipiens* complex taxa examined here as well as the outgroup, *Cx*. *torrentium*. Weighted estimates were always larger than unweighted estimates. Not surprisingly, the highest values were observed between the *Cx*. *pipiens* complex taxa and the *Cx*. *torrentium* sample. Among the taxa within the *Cx*. *pipiens* complex, the highest unweighted F_st_ value was between *Cx*. *quinquefasciatus* and *Cx*. *pipiens* f. pipiens (0.2967). With weighted F_st_ values, the highest was between *Cx*. *quinquefasciatus* and *Cx*. *pipiens* f. molestus (0.6415). The lowest estimated values were between the two *Cx*. *pipiens* forms (unweighted = -0.1026, weighted = 0.0276).

**Table 4:** Pairwise F_st_ values between taxa. Given are both unweighted and weighted measures for each pair of taxa (excluding known hybrid samples). Taxonomic designations were determined prior to this study (see text for more details).

TAXON	*Cx*. *torrentium*	*Cx*. *pipiens pallens*	*Cx*. *pipiens* f. pipiens	*Cx*. *pipiens* f. molestus	*Cx*. *quinquefasciatus*	
***Cx*. *torrentium***		0.5744	0.2752	0.4989	0.4065	Unweighted F_st_ Values
***Cx*. *pipiens pallens***	0.7468		0.1594	0.3166	0.2585	
***Cx*. *pipiens* f. pipiens**	0.5724	0.3192		-0.1026	0.2967	
***Cx*. *pipiens* f. molestus**	0.7167	0.5050	0.0276		0.2656	
***Cx*. *quinquefasciatus***	0.7827	0.5475	0.5634	0.6415		
	Weighted F_st_ Values					

Pairwise unweighted and weighted estimates of F_st_ between the six designated geographic populations of *Cx*. *quinquefasciatus* are given in Table 5. Again, the weighted estimates were always larger than the unweighted estimates. For both estimate types, the highest values were observed between samples from Nigeria and Samoa (unweighted = 0.1233, weighted = 0.2387). For unweighted F_st_ values, the lowest estimate was between samples from Brazil and North America, including the Caribbean (-0.2329). The lowest estimated weighted F_st_ value was between Brazilian and east Asian samples (-0.0668).

**Table 5:** Pairwise F_st_ values between *Cx*. *quinquefasciatus* populations. Given are both unweighted and weighted measures for each pair of populations. Population designations were assigned based on collection location (see text for more details).

POPULATION	east Asia	Samoa	Hawaii	North America & Caribbean	Brazil	Nigeria	
**east Asia**		0.0514	0.0593	0.0051	-0.2280	0.0161	Unweighted F_st_ Values
**Samoa**	0.1611		0.0168	0.1016	-0.0514	0.1233	
**Hawaii**	0.1326	0.1026		-0.0265	-0.1741	0.0578	
**North America & Caribbean**	0.1104	0.2277	0.0327		-0.2329	0.0342	
**Brazil**	-0.0668	0.1622	0.0234	-0.0638		-0.1288	
**Nigeria**	0.1100	0.2387	0.1626	0.1389	0.0497		
	Weighted F_st_ Values						

### Phylogenetic analysis

**Fig 4:**
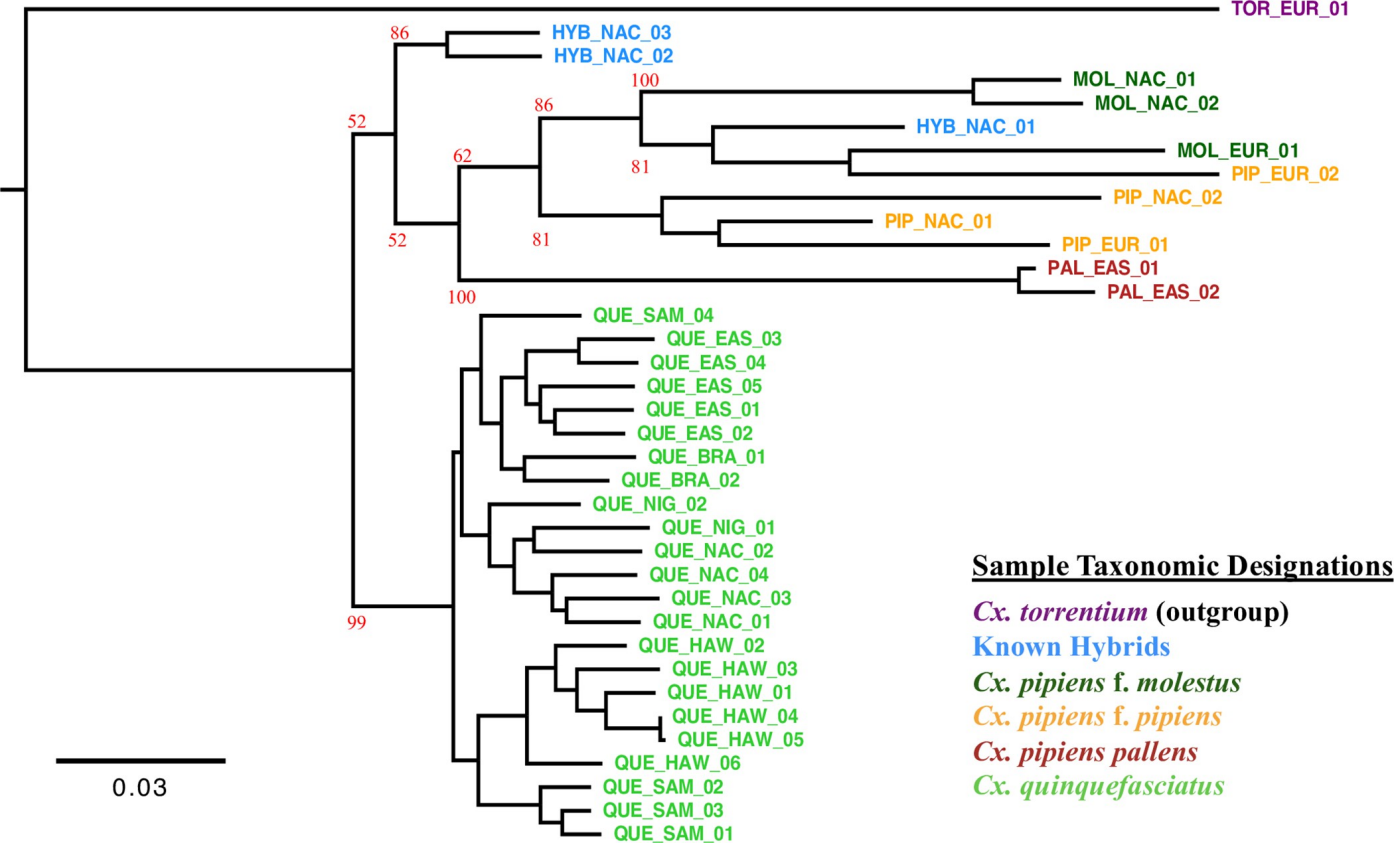
Maximum-likelihood phylogeny of samples. A maximum-likelihood analysis of all samples using a transversional model of mutation with a gamma distribution of rate heterogeneity TVM + Γ; Tavaré, 1986 [71]. 100 bootstrap replicates of the analysis were performed and the bootstrap support for major nodes are shown in red. The colors correspond to the different taxonomic designations.

The dataset for our phylogenetic analysis consisted of 1,735 unlinked 4-fold synonymous SNPs, all of which were present in at least 75% of the samples. The evaluation of models of nucleotide sequence evolution indicated that a transversional model of mutation with a gamma distribution of rate heterogeneity best fit the data (TVM + Γ) [71]. As expected, the outgroup species *Cx*. *torrentium* was clearly distinct from the samples of the *Cx*. *pipiens* complex (Fig 4). The *Cx*. *quinquefasciatus* samples also clustered with high confidence and overall, clustering in our phylogeny recapitulated the results of the PCAs and ADMIXTURE analyses.

### Presence of variants potentially conferring insecticide resistance

After reviewing the literature, we investigated the presence and frequency of seven single nucleotide polymorphisms that have been shown to correlate with insecticide resistance in the *Cx*. *pipiens* complex (Table 6). Interestingly, all presumptive resistance-associated alleles were present among the samples we examined. For one of these sites, R213, found in gene CYP6BZ2 (cytochrome P450 6BZ2), there are two allelic changes that are associated with resistance (R213L and R213Q). No sample had a copy of both resistance-associated alleles, however, only four samples were homozygous for the susceptible nucleotides at this site. Of the eight possible resistance-associated alleles at seven sites, only those in the cytochrome P450, 6BZ2 gene were observed at frequencies greater than 20% (T:41.7%, A:33.3%) across all surveyed mosquito samples. All other resistance-associated alleles were found at lower frequencies than their alternative, susceptible allele.

**Table 6:** Summary of Insecticide Resistance-Associated Allele Frequencies. Given are the genomic position of examined SNPs that were previously found to correlate with resistance (chromosome and base position), gene ID (in the annotated *Cx*. *quinquefasciatus* genome), gene name, amino acid change, number of chromosomes examined (e.g., # of samples the variant is called at position * 2), and the frequencies of the susceptible and resistant alleles.

Chromosome	Position	Gene ID	Gene Name	Amino Acid Change	Number of Chromosomes Examined (n = 72)	Susceptible Allele Frequency	Resistance Allele Frequency	Reference
supercont3.35	864866	CPIJ002538	CYP6AG12: cytochrome P450 6AG12	H293L	72	A:0.986	T:0.014	[72]
supercont3.104	245887	CPIJ005956	CYP6BZ2: cytochrome P450 6BZ2	R213L, R213Q	72	G:0.250	T:0.417, A:0.333	[72]
supercont3.106	33980	CPIJ006034	ACE-1: acetylcholinesterase	G247S	56	G:0.929	A:0.071	[73]
supercont3.106	46973	CPIJ006034	ACE-1: acetylcholinesterase	T682A	72	A:0.917	G:0.083	[73]
supercont3.196	232328	CPIJ008566	CYP6Z15: cytochrome P450 6Z15	E243A	70	A:0.857	C:0.143	[72]
supercont3.228	585169	CPIJ009085	CYP6AG13: cytochrome P450 6AG13	N211D	52	A:0.827	G:0.173	[72]
supercont3.510	164957	CPIJ014218	CYP9M10: cytochrome P450 9M10	F245I	66	A:0.864	T:0.136	[72]

## Discussion

We present evidence that targeted gene enrichment in *Culex* mosquitoes is an effective way to substantially increase the amount of sequence data from non-repetitive genomic regions of known function (i.e.; coding sequences). We also show that this data can be used to survey a large number of segregating genetic sites from across the genomes of several *Culex pipiens* complex samples. Use of these sites allowed us to successfully examine taxonomic relationships, population structure, and patterns of admixture in these mosquitoes, and recovered similar patterns of population differentiation observed after the analyses of thousands of specimens at 7–12 microsatellite loci [10,12–14,31]. We also showed enrichment approach has utility for surveying the presence and frequency of alleles known to correlate with insecticide resistance.

Perhaps not surprisingly, the genetic reads derived from *Cx*. *quinquefasciatus* samples mapped the best to the reference genome, while the outgroup sample, *Cx*. *torrentium*, mapped the poorest (S3 Table). Using just the *Cx*. *quinquefasciatus* samples to look at the relationship between number of raw reads generated and the number of successfully mapped reads, we observed a small but significant, positive trend (S5 Fig). This suggests that a greater depth of sequencing is advisable, as this would increase the number of reads per sample, but there are likely other factors to consider. These may include the age of the sample (and corresponding DNA degradation), and the relative taxonomic distance from the reference [74]. In the latter case, the number of variants which will be useful in downstream analyses may not be greatly improved by a greater depth of sequencing.

In our clustering analysis using principal components, we observed the greatest genetic distinction between the *Cx*. *quinquefasciatus* samples and those of the other taxa (Fig 1). Interestingly, the samples of *Cx*. *quinquefasciatus* clustered more tightly than these other samples when considered collectively. This result was also seen in our DAPC (S3B Fig). The more loosely defined cluster for non-*Cx*. *quinquefasciatus* samples likely reflects the greater amount of genetic divergence harbored within these taxa, and may support the unique taxonomic designations attributed to them. However, we also observed high levels of genetic diversity within these taxa, particularly *Cx*. *pipiens* f. pipiens and *Cx*. *pipiens pallens* (Table 2). It remains to be determined how much of this is true biological diversity, and how much could be an artifact of reference-based mapping biases.

We also observed two primary genetic groups in our ADMIXTURE analysis (Fig 2), with K = 2 being the best supported (S2 Fig). As with our PCA, these correspond to a *Cx*. *quinquefasciatus* cluster and a cluster with all other samples. In both the PCA and ADMIXTURE analysis, the hybrid samples showed the expected mixture of lineages.

While the best supported K value in these analyses indicate the number of confidently discreet taxa or populations, examinations of additional K values can provide important insights into patterns of more nuanced genetic divergences among the samples, as well as indicate samples that may be admixed. Interestingly, in our ADMIXTURE analysis at K = 3, the *Cx*. *quinquefasciatus* samples became split between a Hawaiian and Samoan group and the rest of the samples. This was somewhat surprising given the patterns of clustering observed in the PCA, which differentiated *Cx*. *pipiens pallens* from the other taxa along the second axis. In the ADMIXTURE analysis, *Cx*. *pipiens pallens* only became distinct at K = 5. These differences may reflect differences between the non-parametric approach of a PCA versus the approach of an ADMIXTURE analysis, which utilizes both allele frequency and ancestry fraction parameters [55].

When we examined clustering in just the *Cx*. *quinquefasciatus* samples, we again observe the greatest differences between the Hawaiian and Samoan samples and everything else (Figs 1 & 3). However, for both our DAPC and ADMIXTURE analysis, K = 1 was the best supported. This is not surprising given that these represent a single taxon with the potential for high rates of inter-population gene flow. Considering patterns of genetic diversity within *Cx*. *quinquefasciatus* populations, the east Asian samples harbored the highest mean number of heterozygous sites and a correspondingly high π value (Table 3). This recapitulates previous examinations of genetic diversity in this species [10]. The lowest genetic diversity was present in the Brazilian samples, which may indicate a relatively recent colonization of South America.

In the quantitative examination of taxonomic differentiation, weighted F_st_ values were always higher than unweighted values (Table 4). Not surprisingly, the greatest F_st_ values observed were between the taxa in the species complex and the outgroup, *Cx*. *torrentium* (Table 4). Interestingly, among taxa in the species complex, the highest unweighted value was observed between *Cx*. *pipiens* f. molestus and *Cx*. *pipiens pallens*, whereas for weighted values it was between *Cx*. *pipiens* f. molestus and *Cx*. *quinquefasciatus*. The distinctiveness of the *Cx*. *pipiens* f. molestus samples from these two taxa is also observed in the principal component analysis (Fig 1). As expected, the lowest weighted and unweighted F_st_ values are both for the comparison of the two forms of *Cx*. *pipiens*.

Within *Cx*. *quinquefasciatus*, the greatest genetic differentiation was between the samples from Nigeria and those from Samoa (Table 5). This may reflect their relative geographic distance from one another and the corresponding decrease in genetic exchange. However, other factors such as differential selection could also play a role in generating the genetic divergence observed between African and Samoan populations of *Cx*. *quinquefasciatus* [75].

In both examinations of taxonomic differentiation using F_st_ values, the number of samples per population being compared was small (Tables 2 and 3). Such small samples sizes can artificially inflate F_st_ estimates [76,77]. However, the large number of variants used in these analyses (916 for all *Cx*. *pipiens* complex samples, and 540 for the *Cx*. *quinquefasciatus* samples only), should have minimized such effects [78]. Nonetheless, it is possible our estimates of F_st_ may not accurately reflect the levels of genetic differentiation which exists between specific populations within the *Cx*. *pipiens* mosquito complex. In the future, analysis of more samples could address this question.

As expected, in the taxonomic analysis the outgroup sample *Cx*. *torrentium* was distinct from the other samples (Fig 4). Within the samples of the *Cx*. *pipiens* complex there are two major clades comprised of the *Cx*. *quinquefasciatus* specimens and everything else. The *Cx*. *quinquefasciatus* clade was well supported (99/100 bootstrap support), whereas the second clade was more poorly supported (52/100 bootstrap support). This was likely due to the presence of the hybrid specimens from North America. Of note, the samples of *Cx*. *pipiens* f. pipiens and *Cx*. *pipiens* f. molestus do not form monophyletic clades in this analysis. This may reflect the low level of genetic differentiation between the two taxa, combined with documented genetic exchange between them [14,25,42,79].

Our assessment of insecticide resistance-associated alleles revealed the presence of all identified variants in at least one of the sequenced samples. This points to the ubiquity and maintenance of these alleles in the *Cx*. *pipiens* complex and underscores the importance of careful insecticide resistance management [80]. However, it should be noted that the individual mosquitoes used here were not assayed for resistance to any insecticide, and therefore the presence of these alleles cannot be explicitly associated with resistance.

Another consideration regarding resistance-associated alleles (and observed genetic variation more broadly), is the extent to which the same derived mutation may have arisen independently in multiple complex populations (‘genetic homoplasy’). We have assumed a single origin for all examined genetic variation, but such an assumption is unlikely to be true across such a large number of segregating sites. If there is extensive homoplasy in the data examined here, this would likely obscure patterns of population clustering and taxonomic differentiation [81]. Considering homoplasy is of particular importance for mutations that may confer a fitness advantage, such as those related to insecticide resistance [82].

Interestingly, all but one of the resistance-associated variants we surveyed were segregating at low frequencies (< 20% of the samples; Table 6). This suggests there may be counter-acting fitness costs to harboring these variants. Indeed, there are known fitness costs associated with mutations in the acetylcholinesterase gene in the absence of strong selection from insecticide exposure [83,84], and such costs may be extended to cytochrome P450 mutations more broadly [85]. The inclusion of 353 genes in our baits assay that could potentially evolve to confer insecticide resistance (i.e., P450s, alpha and beta esterases, sodium channel genes, and acetylcholinesterase genes) means that in the future the methodologies described here could be used to survey known genetic variation that contributes to resistance. Furthermore, these methods could also be a cost-effective way to screen for novel mutations associated with insecticide resistance in these genes.

## Conclusions and future directions

In conclusion, the described bait-based assay is a powerful tool for improving sequencing efficiency and for addressing phylogenomic questions at multiple scales, including questions of taxonomic differentiation and population structure, across the *Cx*. *pipiens* complex. It can also be used to uncover the presence and extent of gene flow among populations and admixture. Furthermore, the utility of the data that can be generated using these baits is likely to expand. For example, it will be possible to investigate specific evolutionary drivers of taxonomic differentiation such as drift or selection. Of particular interest will be the identification of variation in specific genes contributing to the extensive ecological and behavioral differences observed among the *Cx*. *pipiens* complex taxa.

## Supporting information

S1 Table Annotated genes used in bait design. (XLSX)

S2 Table Sample information. (XLSX)

S3 Table Sample sequencing and mapping statistics. (XLSX)

S4 Table Individual genetic diversity observations for all samples. (XLSX)

S5 Table Individual genetic diversity observations for just *Cx*. *quinquefasciatus* samples. (XLSX)

S6 Table Presence of insecticide-resistance associated alleles by sample. (XLSX)

S1 Fig Genomic location of regions (colored bars) covered by the capture baits utilized in this study. *Culex* mosquitoes have three chromosomes, which do not have centromeres. The gene classification is indicated in the key at the lower right corner of the figure.
(PDF)

S2 Fig Variant quality distributions. Filtering thresholds are indicated by the red vertical bars. See text for more details.
(PDF)

S3 Fig Results of our DAPC a) BIC scores for K values 1–18, for all Cx. pipiens complex samples. b) Density plot considering the first discriminant function. The Cx. quinquefasciatus sample cluster is indicated on the right in red, and the cluster for all other complex samples is indicated on the left in blue. Each hash along the horizontal axis represents one sample. c) Genotype composition plot (compoplot) indicating the attributed probabilities of each sample to a cluster. Cx. quinquefasciatus samples are indicated in red and all other complex samples are indicated in blue. d) BIC scores for K values 1–12, for just Cx. quinquefasciatus samples.
(PDF)

S4 Fig Admixture CV results. a) all *Cx*. *pipiens* complex samples b) just *Cx*. *quinquefasciatus* samples
(PDF)

S5 Fig Correlation between number of raw reads sequenced and the percent of these reads that subsequently mapped to the reference genome (only *Cx*. *quinquefasciatus* samples). (PDF)

## References

[1] Aardema ML, Olatunji SK, Fonseca DM. The enigmatic *Culex pipiens* (Diptera: Culicidae) species complex: phylogenetic challenges and opportunities from a notoriously tricky mosquito group. Annals of the Entomological Society of America. 2022; 115:95–104.

[2] Kramer LD, Styer LM, Ebel GD. A global perspective on the epidemiology of West Nile virus. Annual Review of Entomology. 2008; 53:61–81. doi: 10.1146/annurev.ento.53.103106.093258 17645411

[3] Eiden M, Gil P, Ziegler U, Rakotoarivony I, Marie A, Frances B, et al. Emergence of two Usutu virus lineages in *Culex pipiens* mosquitoes in the Camargue, France, 2015. Infection, Genetics and Evolution. 2018; 61:151–4. doi: 10.1016/j.meegid.2018.03.020 29592838

[4] Bataille A, Cunningham AA, Cedeno V, Cruz M, Eastwood G, Fonseca DM, et al. Evidence for regular ongoing introductions of mosquito disease vectors into the Galápagos Islands. Proceedings of the Royal Society B: Biological Sciences. 2009; 276:3769–3775. doi: 10.1098/rspb.2009.0998 19675009 PMC2817279

[5] Paxton EH, Camp RJ, Gorresen PM, Crampton LH, Leonard DL, VanderWerf EA. Collapsing avian community on a Hawaiian island. Science Advances. 2016; 2:e1600029. doi: 10.1126/sciadv.1600029 27617287 PMC5014469

[6] McClure KM, Fleischer RC, Kilpatrick AM. The role of native and introduced birds in transmission of avian malaria in Hawaii. Ecology. 2020; 101:e03038. doi: 10.1002/ecy.3038 32129884 PMC7332373

[7] Harvey-Samuel T, Ant T, Sutton J, Niebuhr CN, Asigau S, Parker P, et al. *Culex quinquefasciatus*: status as a threat to island avifauna and options for genetic control. CABI Agriculture and Bioscience. 2021; 2:1–21.

[8] Gippet JM, Liebhold AM, Fenn-Moltu G, Bertelsmeier C. Human-mediated dispersal in insects. Current Opinion in Insect Science. 2019; 35:96–102. doi: 10.1016/j.cois.2019.07.005 31479895

[9] Gloria-Soria A, Ayala D, Bheecarry A, Calderon-Arguedas O, Chadee DD, Chiappero M, et al. Global genetic diversity of *Aedes aegypti*. Molecular Ecology. 2016; 25:5377–95. doi: 10.1111/mec.13866 27671732 PMC5123671

[10] Fonseca DM, Smith JL, Wilkerson RC, Fleischer RC. Pathways of expansion and multiple introductions illustrated by large genetic differentiation among worldwide populations of the southern house mosquito. American Journal of Tropical Medicine and Hygiene. 2006; 74:284–289. 16474085

[11] Dine DLV. Mosquitoes in Hawaii. Hawaii Agricultural Experimental Station Bulletin. 1904; 6:1–30.

[12] Fonseca DM, Keyghobadi N, Malcolm CA, Mehmet C, Schaffner F, Mogi M, et al. Emerging vectors in the *Culex pipiens* complex. Science. 2004; 303:1535–1538. doi: 10.1126/science.1094247 15001783

[13] Fonseca DM, Smith JL, Kim HC, Mogi M. Population genetics of the mosquito *Culex pipiens* pallens reveals sex-linked asymmetric introgression by *Culex quinquefasciatus*. Infection, Genetics and Evolution. 2009; 9:1197–1203. doi: 10.1016/j.meegid.2009.06.023 19584006 PMC2787783

[14] Kothera L, Zimmerman EM, Richards CM, Savage HM. Microsatellite characterization of subspecies and their hybrids in *Culex pipiens* complex (Diptera: Culicidae) mosquitoes along a north-south transect in the central United States. Journal of Medical Entomology. 2009; 46:236–248. doi: 10.1603/033.046.0208 19351074

[15] Goodwin S, McPherson JD, McCombie WR. Coming of age: ten years of next-generation sequencing technologies. Nature Reviews Genetics. 2016; 17:333–351. doi: 10.1038/nrg.2016.49 27184599 PMC10373632

[16] Kulkarni P, Frommolt P. Challenges in the setup of large-scale next-generation sequencing analysis workflows. Computational and Structural Biotechnology Journal. 2017; 15:471–477. doi: 10.1016/j.csbj.2017.10.001 29158876 PMC5683667

[17] Whitfield ZJ, Dolan PT, Kunitomi M, Tassetto M, Seetin MG, Oh S, et al. The diversity, structure, and function of heritable adaptive immunity sequences in the *Aedes aegypti* genome. Current Biology. 2017; 27:3511–3519. doi: 10.1016/j.cub.2017.09.067 29129531 PMC5698160

[18] Black WC, Rai KS. Genome evolution in mosquitoes: intraspecific and interspecific variation in repetitive DNA amounts and organization. Genetics Research. 1988; 51:185–196. doi: 10.1017/s0016672300024289 2901385

[19] Peng C, Qian Z, Xinyu Z, Qianqian L, Maoqing G, Zhong Z, et al. A draft genome assembly of *Culex pipiens pallens* (Diptera: Culicidae) using PacBio sequencing. Genome Biology and Evolution. 2021; 13:evab005. doi: 10.1093/gbe/evab005 33501937 PMC7936019

[20] Campana MG, Hawkins MT, Henson LH, Stewardson K, Young HS, Card LR, et al. Simultaneous identification of host, ectoparasite and pathogen DNA via in-solution capture. Molecular Ecology Resources. 2016; 16:1224–1239. doi: 10.1111/1755-0998.12524 26990246

[21] Cassin-Sackett L, Callicrate TE, Fleischer RC. Parallel evolution of gene classes, but not genes: Evidence from Hawai’ian honeycreeper populations exposed to avian malaria. Molecular Ecology. 2019; 28:568–583. doi: 10.1111/mec.14891 30298567

[22] Quek RZ, Jain SS, Neo ML, Rouse GW, Huang D. Transcriptome-based target-enrichment baits for stony corals (Cnidaria: Anthozoa: Scleractinia). Molecular Ecology Resources. 2020; 20:807–818. 10.1111/1755-0998.13150 PMC7468246 32077619

[23] Itokawa K, Sekizuka T, Maekawa Y, Yatsu K, Komagata O, Sugiura M, et al. High-throughput genotyping of a full voltage-gated sodium channel gene via genomic DNA using target capture sequencing and analytical pipeline MoNaS to discover novel insecticide resistance mutations. PLoS Neglected Tropical Diseases. 2019; 13:e0007818. doi: 10.1371/journal.pntd.0007818 31738756 PMC6886866

[24] Rivero A, Vezilier J, Weill M, Read AF, Gandon S. Insecticide control of vector-borne diseases: when is insecticide resistance a problem?. PLoS Pathogens. 2010; 6:e1001000. doi: 10.1371/journal.ppat.1001000 20700451 PMC2916878

[25] Price DC, Fonseca DM. Genetic divergence between populations of feral and domestic forms of a mosquito disease vector assessed by transcriptomics. PeerJ. 2015; 3:e807. doi: 10.7717/peerj.807 25755934 PMC4349049

[26] Adams MD, Celniker SE, Holt RA, Evans CA, Gocayne JD, Amanatides PG, et al. The genome sequence of *Drosophila melanogaster*. Science. 2000; 287:2185–2195. doi: 10.1126/science.287.5461.2185 10731132

[27] Asgharian H, Chang PL, Lysenkov S, Scobeyeva VA, Reisen WK, Nuzhdin SV. Evolutionary genomics of *Culex pipiens*: global and local adaptations associated with climate, life-history traits and anthropogenic factors. Proceedings of the Royal Society B: Biological Sciences. 2015; 282:20150728. doi: 10.1098/rspb.2015.0728 26085592 PMC4590483

[28] Arensburger P, Megy K, Waterhouse RM, Abrudan J, Amedeo P, Antelo B, et al. Sequencing of *Culex quinquefasciatus* establishes a platform for mosquito comparative genomics. Science. 2010; 330:86–88. doi: 10.1126/science.1191864 20929810 PMC3740384

[29] Giraldo-Calderón GI, Emrich SJ, MacCallum RM, Maslen G, Dialynas E, Topalis P, et al. VectorBase: an updated bioinformatics resource for invertebrate vectors and other organisms related with human diseases. Nucleic Acids Research. 2015; 43:D707–713. doi: 10.1093/nar/gku1117 25510499 PMC4383932

[30] Camacho C. BLAST+ Release Notes. https://www.ncbi.nlm.nih.gov/books/NBK131777/. 2013.

[31] Strickman D, Fonseca DM. Autogeny in *Culex pipiens* complex mosquitoes from the San Francisco Bay Area. The American Journal of Tropical Medicine and Hygiene. 2012; 87:719. doi: 10.4269/ajtmh.2012.12-0079 22869630 PMC3516326

[32] Fonseca DM, Lapointe DA, Fleischer RC. Bottlenecks and multiple introductions: population genetics of the vector of avian malaria in Hawaii. Molecular Ecology. 2000; 9:1803–1814. doi: 10.1046/j.1365-294x.2000.01070.x 11091316

[33] Kruger F. Trim Galore v. 0.4.1 Available from: https://www.bioinformatics.babraham.ac.uk/projects/trim_galore/.

[34] Martin M. Cutadapt removes adapter sequences from high-throughput sequencing reads. EMBnet.journal. 2011; 17:10–12.

[35] Li H. Aligning sequence reads, clone sequences and assembly contigs with BWA-MEM. arXiv preprint arXiv:1303.3997. 2013 Mar 16.

[36] Broad Institute. Picard v. 1.119 Available from: http://broadinstitute.github.io/picard/.

[37] McKenna A, Hanna M, Banks E, Sivachenko A, Cibulskis K, Kernytsky A, et al. The Genome Analysis Toolkit: a MapReduce framework for analyzing next-generation DNA sequencing data. Genome Research. 2010; 20:1297–1303. doi: 10.1101/gr.107524.110 20644199 PMC2928508

[38] Poplin R, Ruano-Rubio V, DePristo MA, Fennell TJ, Carneiro MO, Van der Auwera GA, et al. Scaling accurate genetic variant discovery to tens of thousands of samples bioRxiv, 201178. 2017. doi: 10.1101/201178

[39] Van der Auwera GA, O’Connor BD. Genomics in the Cloud: Using Docker, GATK, and WDL in Terra (1st Edition). 2020. O’Reilly Media.

[40] DePristo M, Banks E, Poplin R, Garimella K, Maguire J, Hartl C, et al. A framework for variation discovery and genotyping using next-generation DNA sequencing data. Nature Genetics. 2011; 43:491–498. doi: 10.1038/ng.806 21478889 PMC3083463

[41] Cingolani P, Platts A, Wang LL, Coon M, Nguyen T, Wang L, et al. A program for annotating and predicting the effects of single nucleotide polymorphisms, SnpEff: SNPs in the genome of *Drosophila melanogaster* strain w1118; iso-2; iso-3. Fly. 2012; 6:80–92. doi: 10.4161/fly.19695 22728672 PMC3679285

[42] Yurchenko AA, Masri RA, Khrabrova NV, Sibataev AK, Fritz ML, Sharakhova MV. Genomic differentiation and intercontinental population structure of mosquito vectors *Culex pipiens* pipiens and *Culex pipiens* molestus. Scientific Reports. 2020; 10:1–13. 32371903 10.1038/s41598-020-63305-z PMC7200692

[43] Li H. Seqtk Toolkit for processing sequences in FASTA/Q formats. GitHub. 2012; 767:69.

[44] Li H, Handsaker B, Wysoker A, Fennell T, Ruan J, Homer N, et al. The Sequence Alignment/Map format and SAMtools. Bioinformatics. 2009; 25:2078–2079. doi: 10.1093/bioinformatics/btp352 19505943 PMC2723002

[45] Patterson N, Price AL, Reich D. Population structure and eigenanalysis. PLoS Genetics. 2006; 2:e190. doi: 10.1371/journal.pgen.0020190 17194218 PMC1713260

[46] Shields DC, Sharp PM, Higgins DG, Wright F. “Silent” sites in *Drosophila* genes are not neutral: evidence of selection among synonymous codons. Molecular Biology and Evolution. 1988; 5:704–716. doi: 10.1093/oxfordjournals.molbev.a040525 3146682

[47] Halligan DL, Eyre-Walker A, Andolfatto P, Keightley PD. Patterns of evolutionary constraints in intronic and intergenic DNA of *Drosophila*. Genome Research. 2004; 14:273–279. doi: 10.1101/gr.1329204 14762063 PMC327102

[48] Andolfatto P. Adaptive evolution of non-coding DNA in *Drosophila*. Nature. 2005; 437:1149–1152. doi: 10.1038/nature04107 16237443

[49] Danecek P, Auton A, Abecasis G, Albers CA, Banks E, DePristo MA, et al. The variant call format and VCFtools. Bioinformatics. 2011; 27:2156–2158. doi: 10.1093/bioinformatics/btr330 21653522 PMC3137218

[50] Purcell S, Neale B, Todd-Brown K, Thomas L, Ferreira MA, Bender D, et al. PLINK: a tool set for whole-genome association and population-based linkage analyses. The American Journal of Human Genetics. 2007; 81:559–575. doi: 10.1086/519795 17701901 PMC1950838

[51] Novembre J, Johnson T, Bryc K, Kutalik Z, Boyko AR, Auton A, et al. Genes mirror geography within Europe. Nature. 2008; 456:98–101. doi: 10.1038/nature07331 18758442 PMC2735096

[52] Pritchard JK, Stephens M, Donnelly P. Inference of population structure using multilocus genotype data. Genetics. 2000; 155:945–59. doi: 10.1093/genetics/155.2.945 10835412 PMC1461096

[53] R Core Team, R. R: A language and environment for statistical computing. https://www.Rproject.org/. 2020; Accessed 22 Jun 2020.

[54] Jombart T, Devillard S, Balloux F. Discriminant analysis of principal components: A new method for the analysis of genetically structured populations. BMC genetics 2010, 11:94. doi: 10.1186/1471-2156-11-94 20950446 PMC2973851

[55] Alexander DH, Novembre J, Lange K. Fast model-based estimation of ancestry in unrelated individuals. Genome Research. 2009; 19:1655–64. doi: 10.1101/gr.094052.109 19648217 PMC2752134

[56] Jombart T, Ahmed I. adegenet 1.3–1: new tools for the analysis of genome-wide SNP data. Bioinformatics. 2011; 27:3070–3071. doi: 10.1093/bioinformatics/btr521 21926124 PMC3198581

[57] Schwarz G. Estimating the dimension of a model. The Annals of Statistics. 1978 Mar 1:461–464.

[58] Alexander DH, Shringarpure SS, Novembre J, Lange K. Admixture 1.3 software manual. Los Angeles: *UCLA Human Genetics Software Distribution*. 2015.

[59] Kopelman NM, Mayzel J, Jakobsson M, Rosenberg NA, Mayrose I. Clumpak: a program for identifying clustering modes and packaging population structure inferences across K. Molecular Ecology Resources. 2015; 15:1179–1191. doi: 10.1111/1755-0998.12387 25684545 PMC4534335

[60] Song K, Li L, Zhang G. Coverage recommendation for genotyping analysis of highly heterologous species using next-generation sequencing technology. Scientific Reports. 2016; 6:1–7. 27760996 10.1038/srep35736 PMC5071758

[61] Nei M, Li WH. Mathematical model for studying genetic variation in terms of restriction endonucleases. Proceedings of the National Academy of Sciences. 1979; 76:5269–5273. doi: 10.1073/pnas.76.10.5269 291943 PMC413122

[62] Nei M. Molecular evolutionary genetics. New York: Columbia Univ. Press; 1987.

[63] Weir BS, Cockerham CC. Estimating F-statistics for the analysis of population structure. Evolution. 1984; 38:1358–1370. doi: 10.1111/j.1558-5646.1984.tb05657.x 28563791

[64] Weir BS, Hill WG. Estimating F-statistics. Annual Review of Genetics. 2002; 36:721–750. doi: 10.1146/annurev.genet.36.050802.093940 12359738

[65] Li H. A statistical framework for SNP calling, mutation discovery, association mapping and population genetical parameter estimation from sequencing data. Bioinformatics. 2011; 27:2987–2993. doi: 10.1093/bioinformatics/btr509 21903627 PMC3198575

[66] Ortiz EM, Palacio-Mejía J. D. vcf2phylip v. 1.5 Available from: https://github.com/edgardomortiz/vcf2phylip/tree/v1.5.

[67] Guindon S, Gascuel O. A simple, fast, and accurate algorithm to estimate large phylogenies by maximum likelihood. Systematic biology. 2003; 52:696–704. doi: 10.1080/10635150390235520 14530136

[68] Darriba D, Taboada GL, Doallo R, Posada D. jModelTest 2: more models, new heuristics and parallel computing. Nature Methods. 2012; 9:772. 10.1038/nmeth.2109 PMC4594756 22847109

[69] Guindon S, Dufayard JF, Lefort V, Anisimova M, Hordijk W, Gascuel O. New algorithms and methods to estimate maximum-likelihood phylogenies: assessing the performance of PhyML 3.0. Systematic Biology. 2010; 59:307–321. doi: 10.1093/sysbio/syq010 20525638

[70] Rambaut A. FigTree v.1.4.4 Comput. Progr. Doc. Distrib. by author, website < http://tree.bio.ed.ac.uk/software/figtree/ > 2018; (accessed 28 December 2018).

[71] Tavaré S. Some probabilistic and statistical problems in the analysis of DNA sequences. Lectures on Mathematics in the Life Sciences. 1986; 17:57–86.

[72] Kothera L, Phan J, Ghallab E, Delorey M, Clark R, Savage HM. Using targeted next-generation sequencing to characterize genetic differences associated with insecticide resistance in *Culex quinquefasciatus* populations from the southern U.S. PLoS One. 2019; 14:e0218397. doi: 10.1371/journal.pone.0218397 31269040 PMC6608931

[73] Zhao M, Dong Y, Ran X, Guo X, Xing D, Zhang Y, et al. Sodium channel point mutations associated with pyrethroid resistance in Chinese strains of *Culex pipiens quinquefasciatus* (Diptera: Culicidae). Parasites & Vectors. 2014; 7:369. 25128988 10.1186/1756-3305-7-369 PMC4261604

[74] Hawkins MT, Hofman CA, Callicrate T, McDonough MM, Tsuchiya MT, Gutiérrez EE, et al. In-solution hybridization for mammalian mitogenome enrichment: Pros, cons and challenges associated with multiplexing degraded DNA. Molecular Ecology Resources. 2016; 16:1173–1188. doi: 10.1111/1755-0998.12448 26220248

[75] Feder JL, Gejji R, Yeaman S, Nosil P. Establishment of new mutations under divergence and genome hitchhiking. Philosophical Transactions of the Royal Society B: Biological Sciences. 2012; 367:461–474. doi: 10.1098/rstb.2011.0256 22201175 PMC3233718

[76] Kalinowski ST. Do polymorphic loci require large sample sizes to estimate genetic distances?. Heredity. 2005; 94:33–36. doi: 10.1038/sj.hdy.6800548 15329660

[77] Morin PA, Martien KK, Taylor BL. Assessing statistical power of SNPs for population structure and conservation studies. Molecular Ecology Resources. 2009; 9:66–73. doi: 10.1111/j.1755-0998.2008.02392.x 21564568

[78] Willing EM, Dreyer C, van Oosterhout C. Estimates of genetic differentiation measured by F(ST) do not necessarily require large sample sizes when using many SNP markers. PLoS One. 2012; 7:e42649. doi: 10.1371/journal.pone.0042649 22905157 PMC3419229

[79] Aardema ML, Vonholdt BM, Fritz ML, Davis SR. Global evaluation of taxonomic relationships and admixture within the *Culex pipiens* complex of mosquitoes. Parasites & Vectors. 2020; 13:1–7. doi: 10.1186/s13071-020-3879-8 31915057 PMC6950815

[80] Dusfour I, Vontas J, David JP, Weetman D, Fonseca DM, Corbel V, et al. Management of insecticide resistance in the major *Aedes vectors* of arboviruses: Advances and challenges. PLoS Neglected Tropical Diseases. 2019; 13:e0007615. doi: 10.1371/journal.pntd.0007615 31600206 PMC6786541

[81] Sanderson MJ, Doyle JJ. Reconstruction of organismal and gene phylogenies from data on multigene families: concerted evolution, homoplasy, and confidence. Systematic Biology. 1992; 41:4–17.

[82] Hawkins NJ, Bass C, Dixon A, Neve P. The evolutionary origins of pesticide resistance. Biological Reviews of the Cambridge Philosophical Society. 2019; 94:135–155. 10.1111/brv.12440 PMC6378405 29971903

[83] Bourguet D, Guillemaud T, Chevillon C, Raymond M. Fitness costs of insecticide resistance in natural breeding sites of the mosquito *Culex pipiens*. Evolution. 2004; 58:128–35. doi: 10.1111/j.0014-3820.2004.tb01579.x 15058725

[84] Rivero A, Magaud A, Nicot A, Vézilier J. Energetic cost of insecticide resistance in *Culex pipiens* mosquitoes. Journal of Medical Entomology. 2011; 48:694–700. doi: 10.1603/me10121 21661333

[85] Hardstone MC, Lazzaro BP, Scott JG. The effect of three environmental conditions on the fitness of cytochrome P450 monooxygenase-mediated permethrin resistance in *Culex pipiens quinquefasciatus*. BMC Evolutionary Biology. 2009; 9:1–3. 19228410 10.1186/1471-2148-9-42 PMC2661048

