# Supplementary figures and images for: A gene-based capture assay for surveying patterns of genetic diversity and insecticide resistance in a worldwide group of invasive mosquitoes
Source: PLoS Negl Trop Dis. 2022 Aug 8;16(8):e0010689. doi: 10.1371/journal.pntd.0010689 (PMC9387926; doi:10.1371/journal.pntd.0010689)

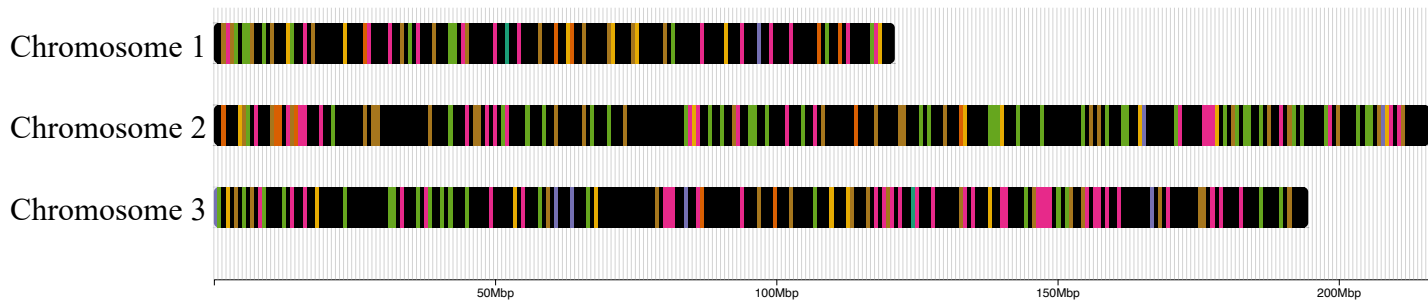

Acetylcholinesterase  
Cadherin  
Esterase  
P450  
Rapidly\_Evolving  
Slow\_Evolving  
Sodium\_Channel

Supplement: S1 Fig — Culex mosquitoes have three chromosomes, which do not have centromeres. The gene classification is indicated in the key at the lower right corner of the figure. (PDF) [file pntd.0010689.s007.pdf]

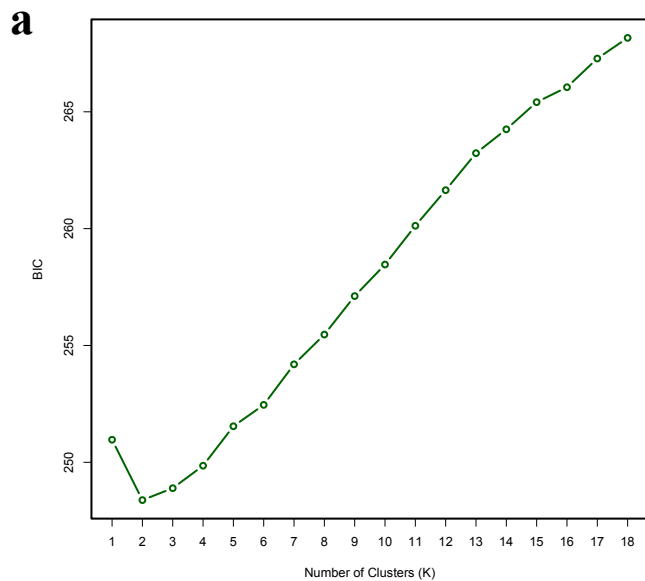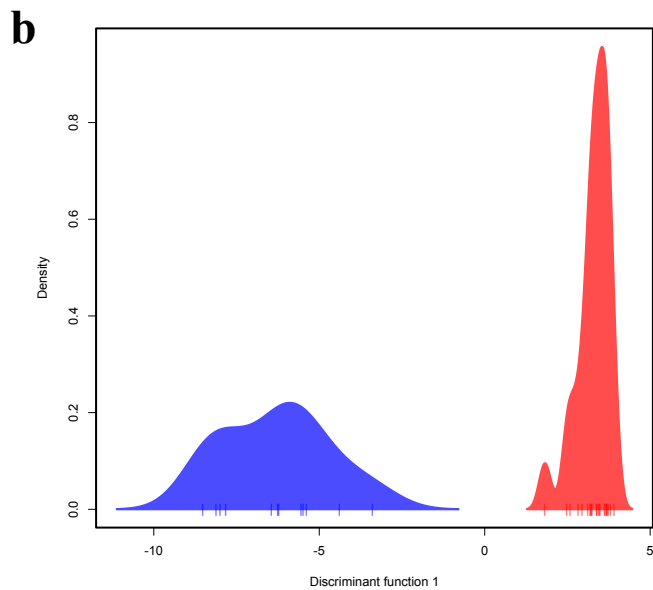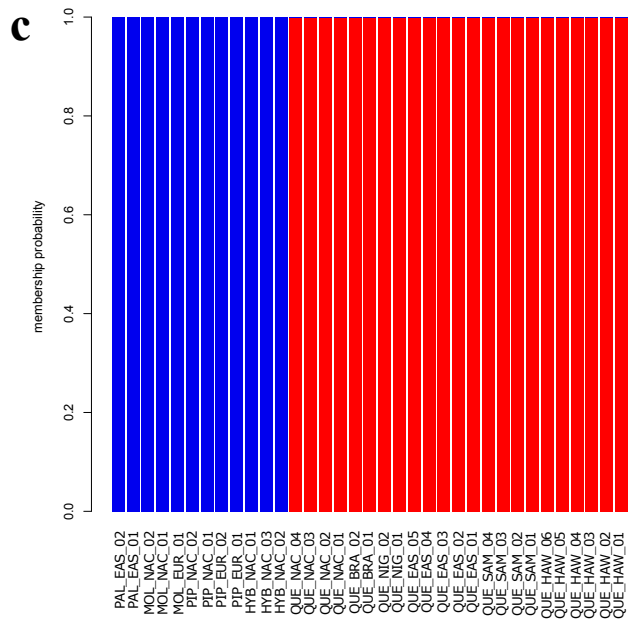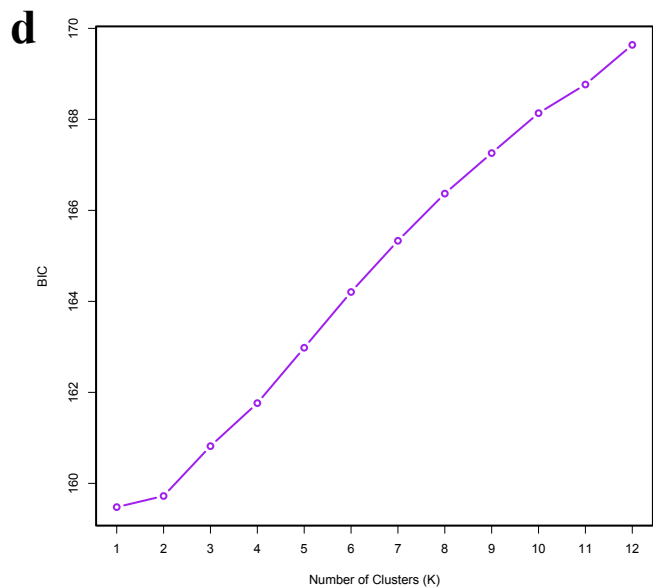

Supplement: S3 Fig — Results of our DAPC a) BIC scores for K values 1–18, for all Cx. pipiens complex samples. b) Density plot considering the first discriminant function. The Cx. quinquefasciatus sample cluster is indicated on the right in red, and the cluster for all other complex samples is indicated on the left in blue. Each hash along the horizontal axis represents one sample. c) Genotype composition plot (compoplot) indicating the attributed probabilities of each sample to a cluster. Cx. quinquefasciatus samples are indicated in red and all other complex samples are indicated in blue. d) BIC scores for K values 1–12, for just Cx. quinquefasciatus samples. (PDF) [file pntd.0010689.s009.pdf]

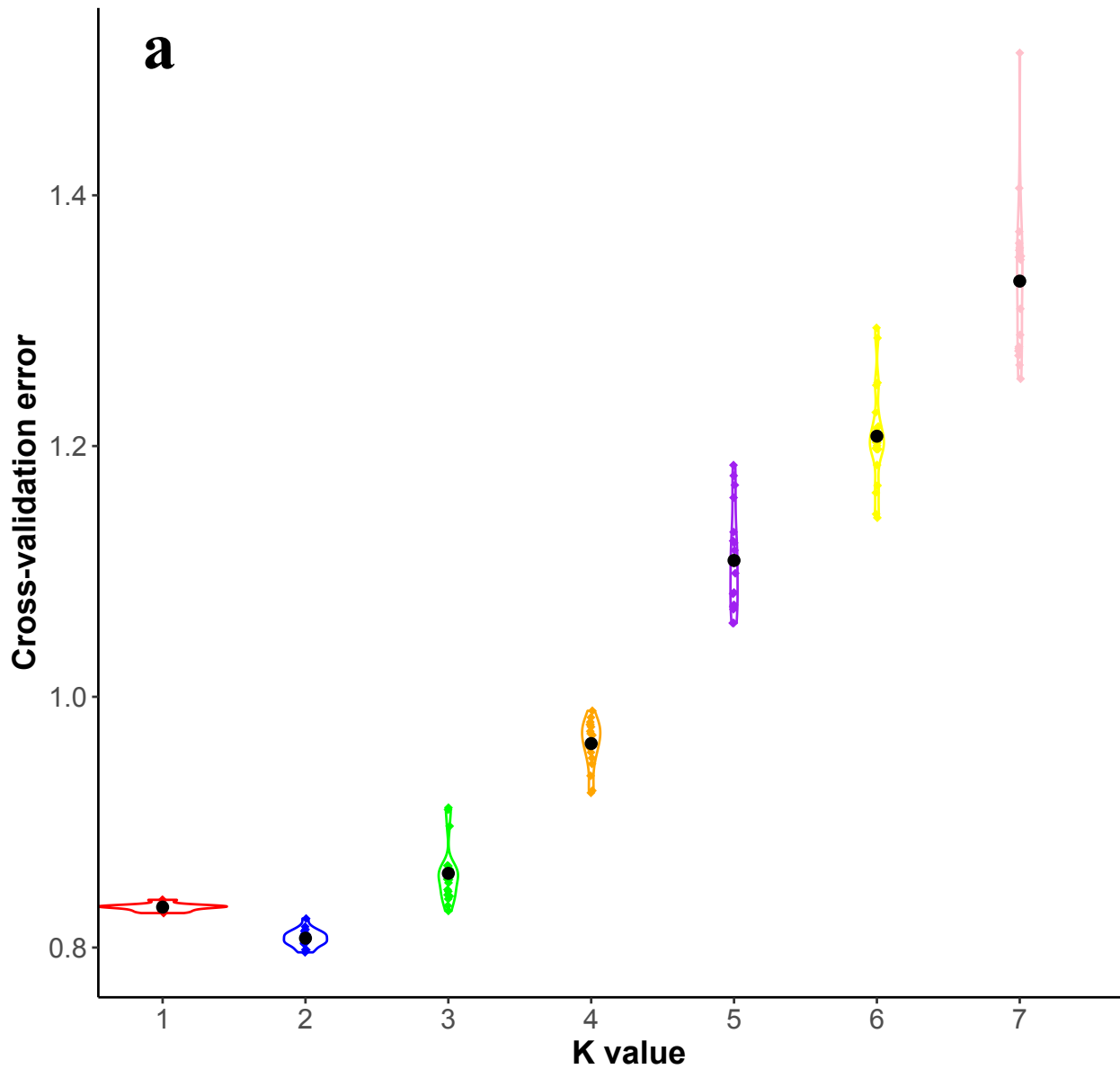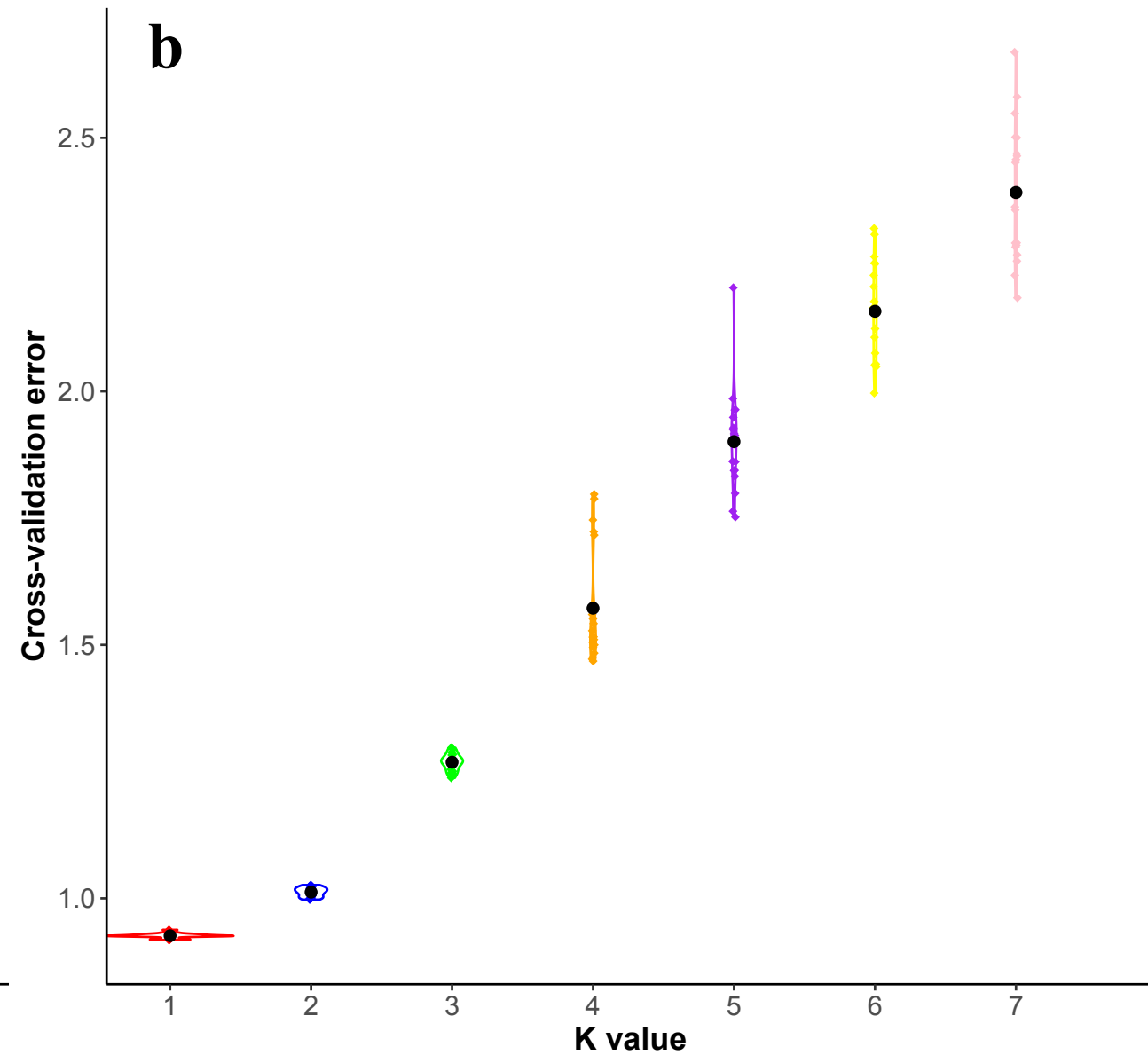

Supplement: S4 Fig — a) all Cx. pipiens complex samples b) just Cx. quinquefasciatus samples (PDF) [file pntd.0010689.s010.pdf]

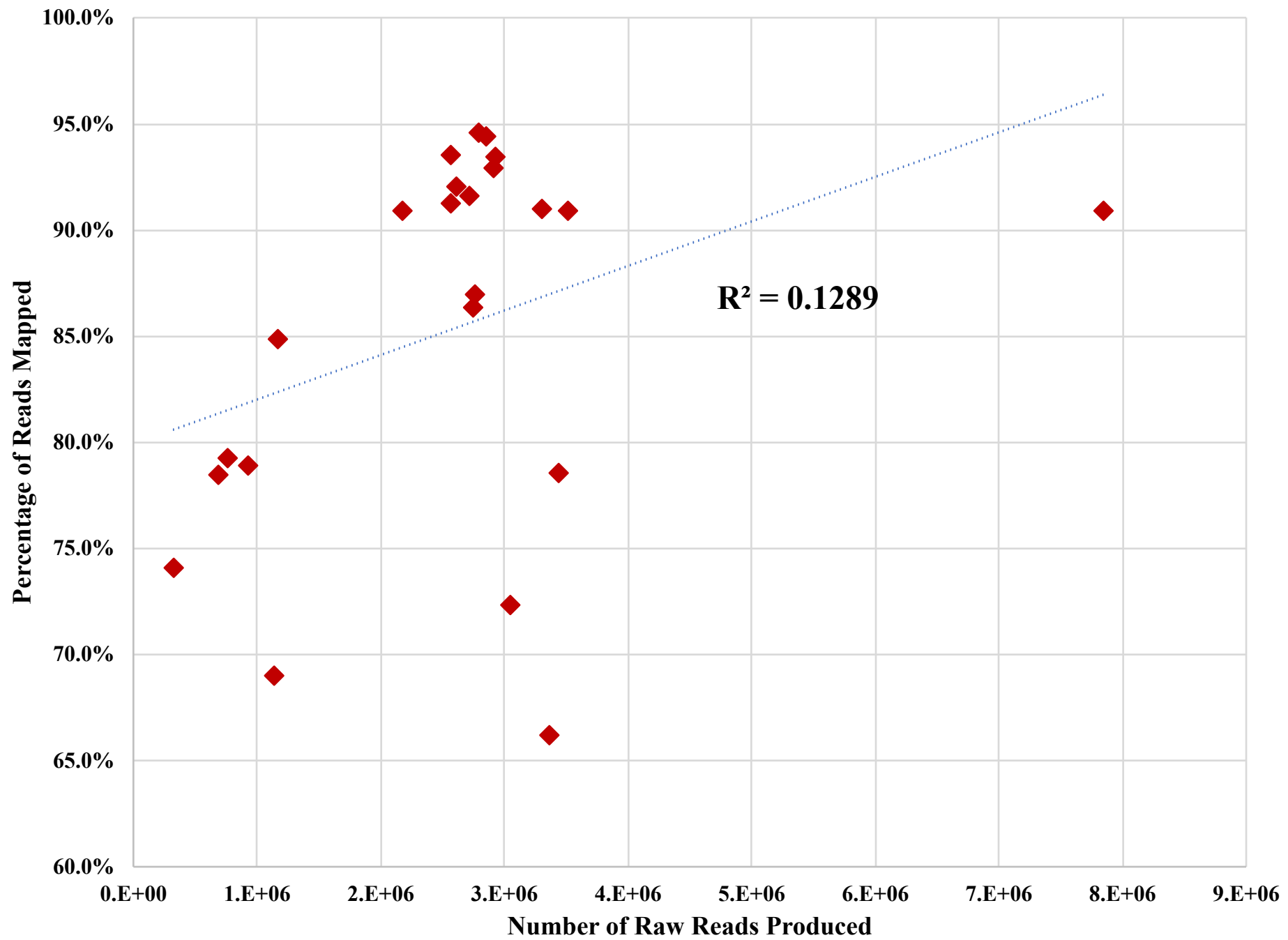

Supplement: S5 Fig — (PDF) [file pntd.0010689.s011.pdf]
