# Supplementary material for: A gene-based capture assay for surveying patterns of genetic diversity and insecticide resistance in a worldwide group of invasive mosquitoes
Source: PLoS Negl Trop Dis. 2022 Aug 8;16(8):e0010689. doi: 10.1371/journal.pntd.0010689 (PMC9387926; doi:10.1371/journal.pntd.0010689)
Supplement: S2 Fig — Filtering thresholds are indicated by the red vertical bars. See text for more details. (PDF) [file pntd.0010689.s008.pdf]

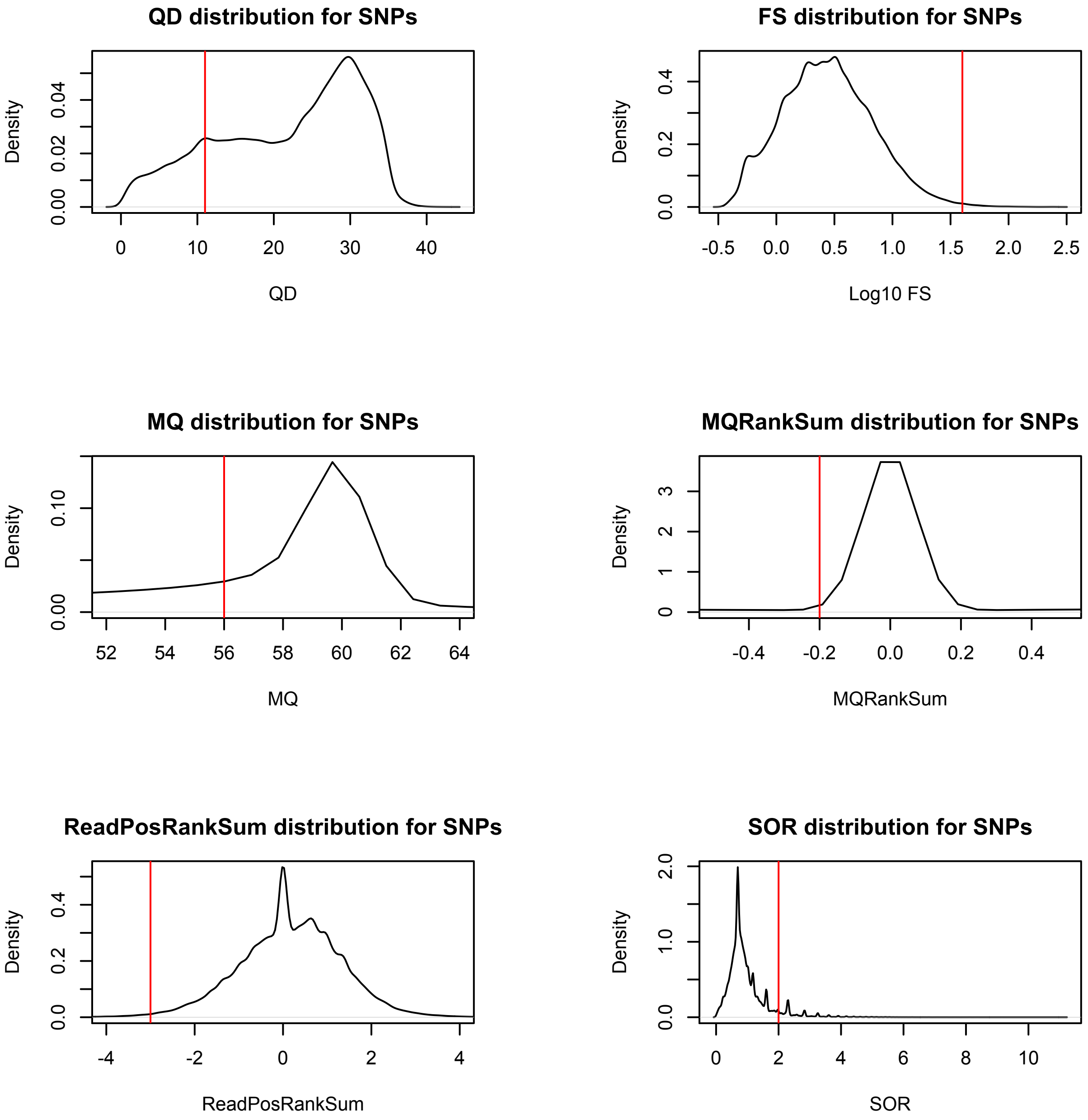
QD distribution for SNPs
Density
QD
0
10
20
30
40
0.00
0.02
0.04
FS distribution for SNPs
Density
Log10 FS
-0.5
0.0
0.5
1.0
1.5
2.0
2.5
0.0
0.2
0.4
MQ distribution for SNPs
Density
MQ
52
54
56
58
60
62
64
0.00
0.10
MQRankSum distribution for SNPs
Density
MQRankSum
-0.4
-0.2
0.0
0.2
0.4
0
1
2
3
ReadPosRankSum distribution for SNPs
Density
ReadPosRankSum
-4
-2
0
2
4
0.0
0.2
0.4
SOR distribution for SNPs
Density
SOR
0
2
4
6
8
10
0.0
1.0
2.0
